# Alpha‐Carbonic Acid Revisited: Carbonic Acid Monomethyl Ester as a Solid and its Conformational Isomerism in the Gas Phase

**DOI:** 10.1002/chem.201904142

**Published:** 2019-11-26

**Authors:** Eva‐Maria Köck, Jürgen Bernard, Maren Podewitz, Dennis F. Dinu, Roland G. Huber, Klaus R. Liedl, Hinrich Grothe, Erminald Bertel, Robert Schlögl, Thomas Loerting

**Affiliations:** ^1^ Institute of Physical Chemistry University of Innsbruck Innrain 52c 6020 Innsbruck Austria; ^2^ Max-Planck-Institut für Chemische Energiekonversion Stiftstraße 34–36 45470 Mülheim an der Ruhr Germany; ^3^ Institute of General, Inorganic and Theoretical Chemistry University of Innsbruck Innrain 80—82 6020 Innsbruck Austria; ^4^ Institute of Materials Chemistry TU Wien Getreidemarkt 9/165 1060 Vienna Austria

**Keywords:** carbonic acids, carbonic acid hemiesters, conformational isomerism, dimerization, isotopic labeling

## Abstract

In this work, earlier studies reporting α‐H_2_CO_3_ are revised. The cryo‐technique pioneered by Hage, Hallbrucker, and Mayer (HHM) is adapted to supposedly prepare carbonic acid from KHCO_3_. In methanolic solution, methylation of the salt is found, which upon acidification transforms to the monomethyl ester of carbonic acid (CAME, HO‐CO‐OCH_3_). Infrared spectroscopy data both of the solid at 210 K and of the evaporated molecules trapped and isolated in argon matrix at 10 K are presented. The interpretation of the observed bands on the basis of carbonic acid [as suggested originally by HHM in their publications from 1993–1997 and taken over by Winkel et al., *J. Am. Chem. Soc*. 2007 and Bernard et al., *Angew. Chem. Int. Ed*. 2011] is inferior compared with the interpretation on the basis of CAME. The assignment relies on isotope substitution experiments, including deuteration of the OH‐ and CH_3_‐ groups as well as ^12^C and ^13^C isotope exchange and on variation of the solvents in both preparation steps. The interpretation of the single molecule spectroscopy experiments is aided by a comprehensive calculation of high‐level ab initio frequencies for gas‐phase molecules and clusters in the harmonic approximation. This analysis provides evidence for the existence of not only single CAME molecules but also CAME dimers and water complexes in the argon matrix. Furthermore, different conformational CAME isomers are identified, where conformational isomerism is triggered in experiments through UV irradiation. In contrast to earlier studies, this analysis allows explanation of almost every single band of the complex spectra in the range between 4000 and 600 cm^−1^.

## Graphical abstract

**We CAME, we saw, we reassigned**: Earlier studies reporting α‐H_2_CO_3_ are revised. In methanolic solution, methylation of the salt is found, which upon acidification transforms to the monomethyl ester of carbonic acid (CAME, HO‐CO‐OCH_3_). Infrared spectroscopy data both of the solid at 210 K and of the evaporated molecules trapped and isolated in argon matrix at 10 K are presented.

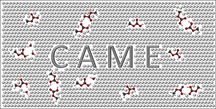

## 1 Introduction

The reactivity of carbonic acid (H_2_CO_3_, CA) towards its ester derivatives has been of interest for more than a century. Starting from the basic formation studies by Hempel and Seidel at the end of the 19th century,^1^ nowadays the interest is more focused towards, for example, its importance in biochemistry^2^ or food chemistry.^3^ Singly esterified carbonic acids are known as hemiesters of carbonic acid (HECAs),^2^ and their salts are known as monoalkyl carbonates (MACs).^4^ The molecule investigated in this work is the methyl hemiester of carbonic acid, which we will refer to as CAME (carbonic acid monomethyl ester) in the following. In biochemistry,^2^ and in food chemistry, that is, carbonated alcoholic beverages,^3^ the focus is on detection and reaction of very low concentrations of carbonic ester derivatives in aqueous solution.^5^ MACs or HECAs are studied from small alkyl esters to quite complex esters, for example, with sugars.^4^

The biological relevance emphasizes the need for simple synthesis and characterization of HECAs as a pure substance. Most synthesis routes have temperatures below 273 K in common. Pure CAME was first synthesized by Hempel and Seidel^1^ in 1898 (by reaction of aqueous CO_2_ with methanol) as a solid that melts between −57 °C and −60 °C. In 1972, Gattow and Behrendt^6^ reported the formation of CAME by using non‐aqueous chemistry, namely the reaction of NaOCH_3_ dissolved in methanol with CO_2_. This hemiester was described as ‘a colorless material that melts at −36 °C′ and was characterized by using infrared spectroscopy. In 2006, Dibenedetto et al.^7^ stated that the isolation of monoalkyl derivatives of H_2_CO_3_ is ′not trivial′. They observed traces of aqueous CAME at room temperature by forming NaOC(O)OCH_3_ (from the reaction of sodium methoxide with CO_2_) and subsequent acidification. Their characterization method of choice was NMR spectroscopy.

In May 2014, one of us, Jürgen Bernard, stated in his Ph.D. thesis that CAME can be synthesized and isolated as a solid by reaction of KHCO_3_ with absolute methanol followed by acidification at cryo‐conditions.^8^ A very similar (but not identical) preparation route was used by Hage, Hallbrucker, and Mayer (HHM) in 1993.^9^ They assigned the resulting solid substance as a polymorph of H_2_CO_3_ on the basis of IR spectroscopy and termed it ′alpha‐carbonic acid′ (α‐H_2_CO_3_). Based on this pioneering work, HHM,^9^, ^10^ Winkel et al.,^11^ and Bernard et al.^12^ assumed in later work that dissolution of KHCO_3_ in methanol followed by acidification leads to α‐H_2_CO_3_. In contrast, ′beta‐carbonic acid′ (β‐H_2_CO_3_) was obtained by HHM by replacing methanol with water as a solvent and by high energy irradiation of CO_2_/H_2_O^13^ mixtures or H‐implantation13b, 14—leading to the claim of polymorphism for H_2_CO_3_. The interpretation of the formation of β‐H_2_CO_3_ remains uncontested. The reinterpretation of all earlier work on α‐H_2_CO_3_ and the polymorphism of H_2_CO_3_ is outlined in the present work, in accordance with the first claim provided in the Ph.D. thesis of Bernard.^8^ The revised interpretation is based on detailed analysis of IR spectra of the solid at cryo‐conditions and single‐molecule IR spectra recorded after sublimation and matrix isolation. These spectra suggest the presence of CAME molecules rather than H_2_CO_3_ molecules as originally envisaged.^12^ In his Ph.D. thesis, Bernard investigated not only CAME but also CAEE—the monoethyl ester of carbonic acid.^8^ The infrared data of solid CAEE as well as individual CAEE molecules isolated in matrix can be found in ref. 15. The reinterpretation of the matrix spectra was also suggested by Reisenauer et al.^16^ in September 2014. In contrast to the present cryo‐study, Reisenauer et al. have studied pyrolysis of dialkyl carbonates at about 1000 K. After isolating the pyrolysis products in argon matrix at 8 K, they identified a product identical to the one found by Bernard.^8^ Both Bernard and Reisenauer et al. identified this product to be carbonic acid monomethyl ester (CAME, HO_2_COCH_3_) rather than H_2_CO_3_. Bernard trapped the gas phase of the sublimed pure solid, while the matrix spectra presented by Reisenauer et al.^16^ contain pyrolysis byproducts such as isobutene, thereby obscuring some spectral ranges. In contrast to our work, Reisenauer et al. also did not provide solid‐state spectra to back up their claim that the solid‐state spectra reported by HHM need reinterpretation.

In the present study, the re‐evaluation of α‐H_2_CO_3_ as CAME is built on four pillars: (i) variation of solvents during both preparation steps; (ii) isotopic shifts in the solid‐state spectra by substitution of the CH_3_ with a CD_3_ group and matching with calculated spectra of species connected by hydrogen bonds; (iii) complete assignment of practically all bands between 4000 and 600 cm^−1^ of matrix isolation IR spectra by considering different CAME conformers, but also CAME dimers and water complexes; and (iv) forced conversion of conformers by irradiation experiments of the molecules trapped in the matrix. The assignment for the single molecules trapped in matrix (item (iii)) is guided by harmonic frequency calculations at the MP2/aug‐cc‐pVTZ level of electronic structure theory and isotopic labeling, leading to the following CAME isotopomers: HO_2_COCD_3_ (CD_3_‐CAME), DO_2_COCH_3_ (OD‐CAME), and HO_2_^13^COCH_3_ (^13^C‐CAME). This strategy puts us in a position to identify minor species present in the matrix besides the two CAME conformers identified by Reisenauer et al.^16^

To learn more about the chemistry of the methyl group in the process leading to the pure solid, the cryo‐technique as employed by HHM^9^, ^10^ was adapted. Specifically, the solvent was evaporated two times rather than just once. HHM were depositing micrometer‐thick sandwiches of alternating glassy layers of acid (e.g., HCl) and base (e.g., KHCO_3_) at 78 K. This sandwich was heated by HHM to induce devitrification (transformation to the supercooled liquid), diffusion, and protonation, after which the solvent was evaporated. In our work, KHCO_3_ was dissolved in methanol, deposited at approximately 80 K, then immediately heated to remove the solvent for the first time. In the next step, the precipitate was cooled to approximately 80 K, and a layer of glassy acid was deposited on top. Heating for a second time induces devitrification, diffusion, and acid–base reaction. After this, the solvent was evaporated again. Evaporating twice (rather than once by HHM) allows for systematic variation of the solvent in the first and second evaporation steps. By using water, methanol, or ethanol and combinations of these for the two evaporation steps, we reveal that the solvent used for the dissolution of the salt in the first step is decisive as to whether H_2_CO_3_,10b CAME, or CAEE^15^ is obtained as the product.

IR bands pertaining to the methyl group in CAME are generally weak. To confidently assign the bands, we not only rely on the absolute calculated frequencies themselves, but also on shifts of bands upon isotope substitution. Furthermore, the band assignment is also guided by matrix irradiation experiments. Upon irradiation of the matrix with UV light, the trapped species can become excited and internal rotation or intramolecular bond cleavage is caused. As the excited molecule or its fragments cannot escape from the cage, different conformers are formed owing to relaxation or recombination. In difference spectra, it is possible to find bands that arise from the same conformational species. The conformer that is formed upon UV irradiation will have bands pointing upwards, whereas the conformer that is depleted will show bands pointing downwards in the difference spectra. Finally, we compare the matrix spectra obtained here with spectra obtained in earlier work after sublimation of the monoethyl hemiester of carbonic acid (CAEE)^15^ and β‐H_2_CO_3_. These strategies allow for an assignment of practically all observed signals in the matrix isolation IR experiments and a clear distinction of bands arising from different monomer conformations and dimers. Based on these procedures, we are even able to determine the ratio of different monomer conformers in the matrix.

## 2 Results and Discussion

### 2.1 Ab initio calculations

The structure of CAME in its solid state is amorphous and thus, not known. The solid‐state spectra reported cannot satisfactorily be explained with the help of ab initio calculations owing to the lack of a well‐defined crystal structure. Accordingly, the assignment for the solid is not based on calculations. Instead, it is provided with the aid of difference spectroscopy and CD_3_‐isotope substitution experiments.

In contrast, matrix isolation spectroscopy is a single‐molecule technique. As isolated molecules are trapped in an inert matrix, such spectra are in general very close to the gas‐phase spectra.^17^ The full width at half maximum (FWHM) of bands in matrix spectra are orders of magnitude smaller than FWHM of bands in solid‐state spectra. Thus, line spectra of individual molecules calculated by ab initio methods in the gas phase are very useful to interpret and assign the observed bands. The static electric field exerted by the inert noble gas argon in matrix isolation spectroscopy is orders of magnitude smaller than the crystal field. Thus, ab initio quantum chemical calculations can directly be used to guide the band assignment of the matrix isolation spectra. Owing to the size of the molecule, we have to rely on the well‐established harmonic approximation. Although the calculated spectra in general match the measured matrix spectra well, there are some small discrepancies even after applying a correction factor to the calculated frequencies. This may arise as a result of anharmonicities and mode–mode coupling effects, which are not included in the harmonic approximation and may cause—depending on the specific mode—a red‐ or blueshift. Furthermore, the matrix cage causes a slight shift of the bands owing to the cage geometry and in some cases a matrix splitting of bands as a result of different cage sites or symmetry reduction of the isolated molecules.^18^

#### 2.1.1 Structures and stabilities of CAME monomers

**Figure 1:**
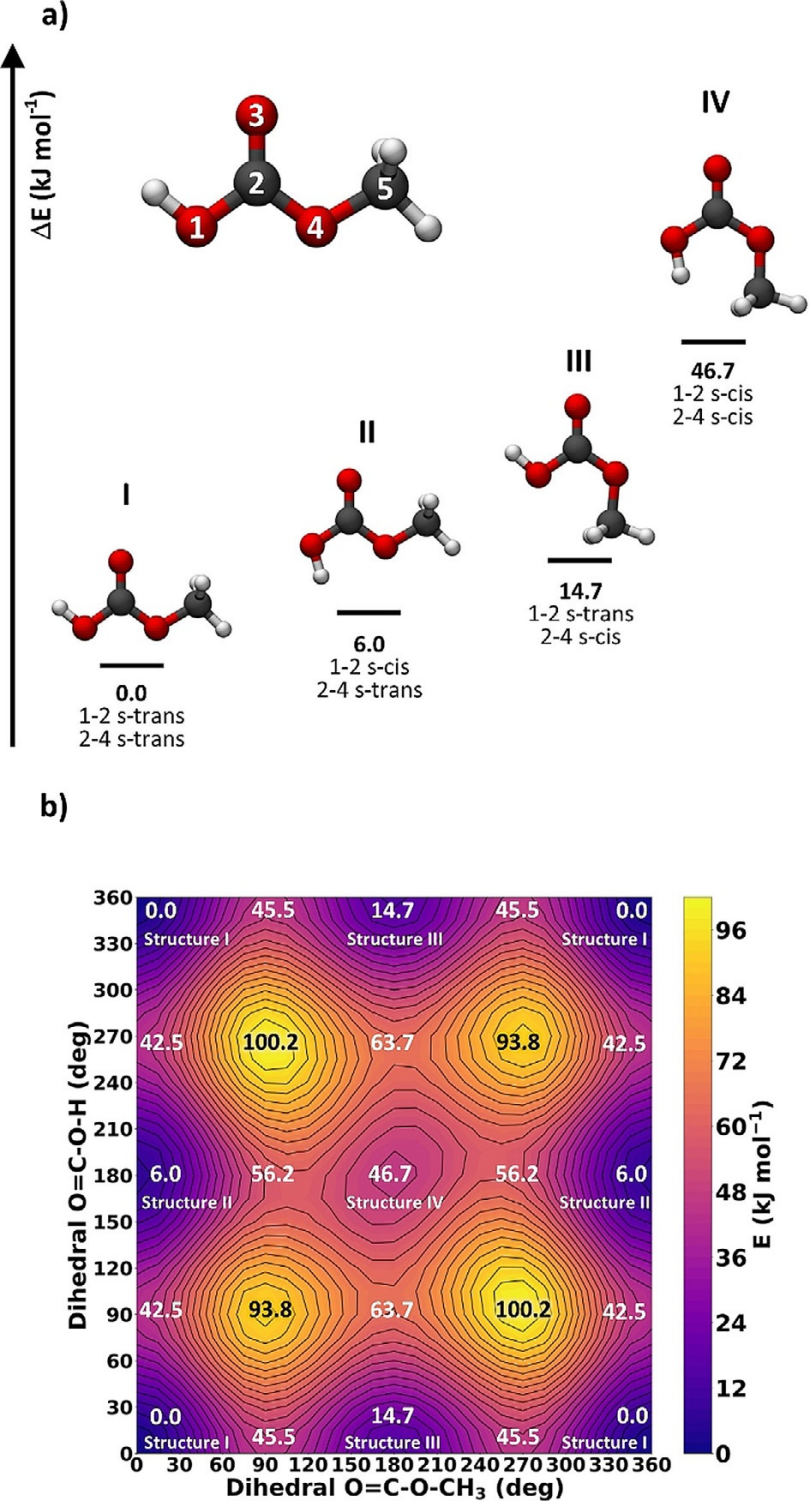
a) Energy minima and stereo‐nomenclature for conformational isomers of CAME according to MP2/aug‐cc‐pVTZ. Energies are electronic energies without zero‐point energy corrections. Atom colors: gray=C, white=H, red=O. b) Potential energy surface for torsional movement of the methyl group (*x* axis) and of the terminal hydrogen atom (*y* axis) as calculated at the MP2/aug‐cc‐pVTZ level of theory.

To assess the conformational space and to estimate the kinetics, the potential energy surface for the torsional movement of the methyl group and the terminal hydrogen atom of the hydroxyl group was calculated with ab initio wave function methods (MP2/aug‐cc‐pVTZ) as depicted in Figure 1. Figure 1 a shows the low‐energy conformations of the CAME molecule and their relative electronic energies. The nomenclature for these conformational isomers is based on an analogy to the nomenclature of 1,3‐butadiene by using the descriptors s‐*cis* and s‐*trans* for the conformation around the single bonds 1–2 and 2–4. Structures I and II are within 6.0 kJ mol^−1^, whereas structure III is slightly higher in energy (+14.7 kJ mol^−1^) and structure IV is energetically rather unfavorable (+46.7 kJ mol^−1^). Explicitly‐correlated coupled cluster single‐point calculations (CCSD(T)‐F12/cc‐pVTZ‐F12) on the MP2/aug‐cc‐pVTZ re‐optimized structures when molecular symmetry is taken into account confirmed the results from the potential energy surface scan and yielded energies of structure I: 0.0 kJ mol^−1^, structure II: 5.8 kJ mol^−1^, and structure III: 14.7 kJ mol^−1^. As these values are very similar to the MP2 ones, convergence in the electronic structure can be assumed.

The experimental low‐temperature conditions make it highly unlikely to detect structures with a relative energy higher than 15–20 kJ mol^−1^ compared with the global energy minimum (structure I)—see also calculations about conversion to structure I during the flight time of the preparation of the matrix, below. Thus, structure IV is from now on neglected.

Similarly, the barriers to rotation for the methyl group and the hydroxyl group as estimated from the potential energy surface (PES) in Figure 1 b are rather substantial, for example, approximately 40 kJ mol^−1^ for the conversion from structure I to structure II and even higher for the conversion to structure III. The remarkable conformational stability and the high torsional barrier of CAME can be rationalized by a minimization of electrostatic and closed‐shell repulsion between the oxygen lone pairs and the carbonyl double bond, which is best realized in the conformation 1–2 s‐*trans* and 2–4 s‐*trans* of structure I. Rotation of the terminal hydrogen or the methyl group to a *cis* conformation is associated with a substantial energy penalty owing to the close vicinity of the oxygen lone pair and the carbonyl double bond, which experience mutual electrostatic and closed‐shell repulsion.

#### 2.1.2 Constitution, stability, and interaction in CAME dimers

Although for carbonic acid (CA), no dimers were found in the matrix,^19^ the situation may be different for CAME. To access whether dimers are viable structures, likely to occur in the matrix, we studied the structures and stabilities of various CAME dimer conformations.

From the three low‐energy conformations of the CAME monomer, six potential CAME dimers could be constructed.

The compositions of the possible dimers are:

dimer1=structureI+structureI

dimer2=structureI+structureIII

dimer3=structureIII+structureIII

dimer4=structureI+structureII

dimer5=structureII+structureIII

dimer6=structureII+structureII

**Figure 2:**
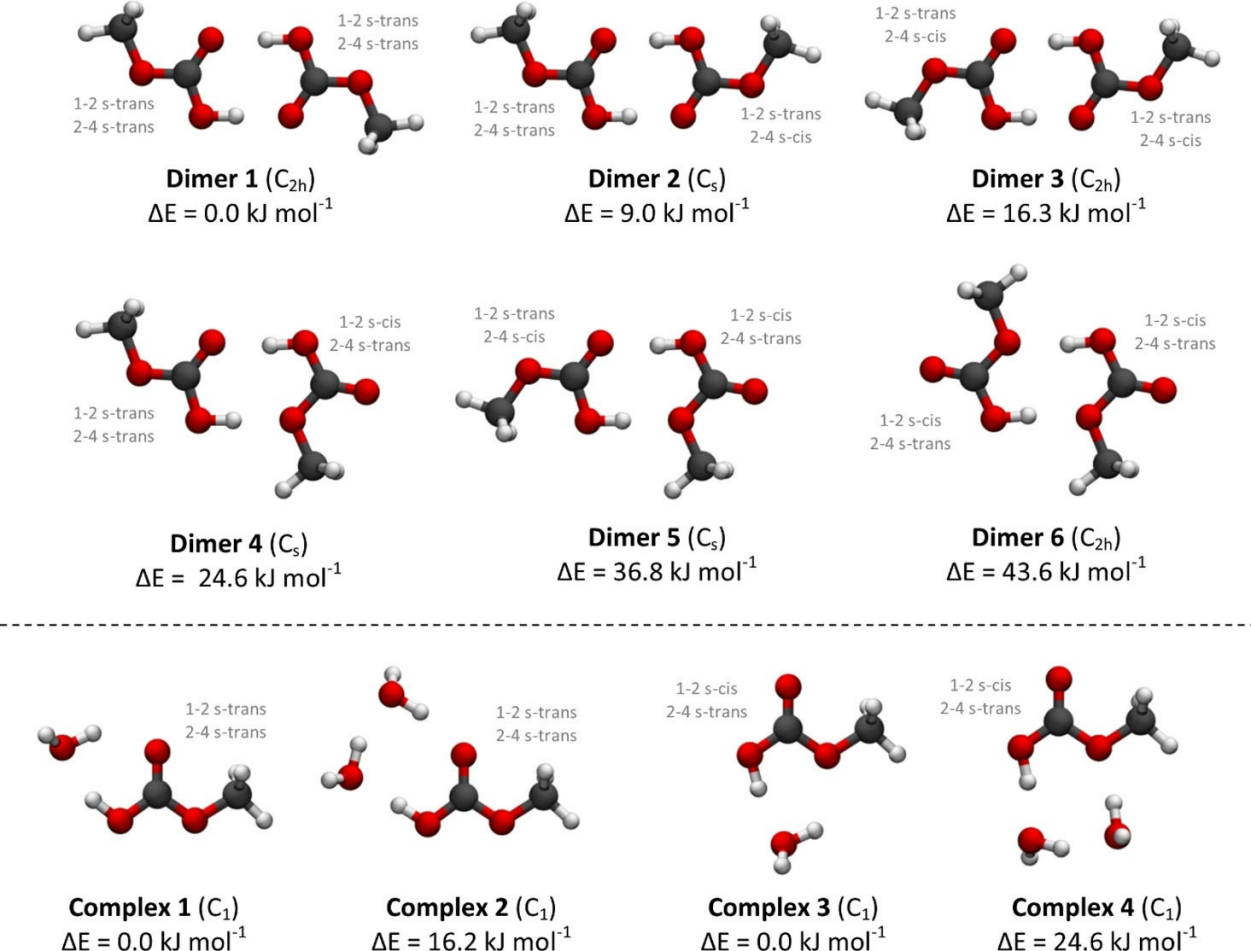
Optimized structures and relative electronic energies (in kJ mol^−1^) of CAME dimers 1, 2, 3 (first row), and 4, 5, 6 (second row), as well as CAME**–**water complexes (last row). All structures were optimized using MP2/aug**‐**cc**‐**pVTZ.

Each dimer is assembled through two hydrogen bonds between the OH⋅⋅⋅O=C of the respective two monomers (Figure 2). All structures were fully optimized with MP2/ aug‐cc‐pVTZ and subsequent CCSD(T)‐F12/cc‐pVTZ‐F12 single‐point calculations, which yielded relative energies as depicted in Table 1. The relative stabilities of these dimers vary by up to 40 kJ mol^−1^. Relative free energies are very similar to the electronic energies (see Table 1). To further assess the characteristics and energetics of these dimers, we calculated dimerization energies and dimerization free energies, that is, how much energy is released when the dimer is formed from two monomers. Interestingly, dimer 3 displays the most negative and thus, most favorable dimerization energy of all six dimers, namely −88.6 kJ mol^−1^. Dimer 2 shows a dimerization energy of −81.5 kJ mol^−1^ and dimer 1 −75.9 kJ mol^−1^, whereas all other structures show higher but still favorable relative energies. When considering dimerization free energies at 210 K, only the formation of dimers 1–3 is exergonic, whereas formation of dimers 4–6 is endergonic. Again, the dimerization is most favored for dimer 3 (−16.7 kJ mol^−1^), a bit less favored for dimer 2 (−10.5 kJ mol^−1^) and dimer 1 (−3.6 kJ mol^−1^), these energies are more favorable than the available thermal energy at 210 K in the classic approximation using *R*⋅*T*, which is 1.7 kJ mol^−1^. To shed light on the interaction and the hydrogen‐bond strength in these dimers, we investigated the interaction energies, that is, the energy gain owing to the interaction of the two monomer fragments (at the geometry of the dimer complex). In contrast, the dimerization energy is the interaction energy plus the energy that is required to distort the optimized monomers to the dimer geometry. Again, dimer 3 shows the most favorable interaction energy (−111.6 kJ mol^−1^) and forms, thus, the strongest hydrogen bonds, followed by dimer 2 (−99.5 kJ mol^−1^) and dimer 1 (−90.3 kJ mol^−1^). This is further supported by structural analyses, where dimer 3 shows the shortest O⋅⋅⋅H bond of 1.559 Å versus 1.596 Å and 1.586 Å, respectively, in dimer 2 and versus 1.619 Å in dimer 1.

**Table 1:** Relative electronic energies of the three low‐energy conformers of the CAME dimers (dimer 1–6), CAME monomers (structure I, II, III) as well as water complexes ((H_2_O)_*x*_—complex 1–4). Structures were fully optimized with MP2/aug‐cc‐pVTZ exploiting the molecular symmetry, coupled cluster calculations (CCSD(T)‐F12/cc‐pVTZ‐F12) are single points on the MP2/aug‐cc‐pVTZ optimized structures. Energies are given in kJ mol^−1^. Free energies are calculated for *T*=210 K and *p*=2×10^−5^ mbar.

Structure	Δ*E*_rel_		Δ*G*_rel_(210 K)	Point group
	MP2	CCSD(T)‐F12 //MP2	CCSD(T)‐F12 //MP2	
dimer 1	0.0	0.0	0.0	*C*_2*h*_
dimer 2	9.0	9.2	7.7	*C_s_*
dimer 3	16.3	16.8	16.0	*C*_2*h*_
dimer 4	24.6	27.3	24.8	*C_s_*
dimer 5	36.8	39.4	36.6	*C_s_*
dimer 6	43.6	48.7	46.5	*C*_2*h*_
monomer				
structure I	0.0	0.0	0.0	*C_s_*
structure II	6.0	5.8	5.4	*C_s_*
structure III	14.7	14.7	14.5	*C_s_*
H_2_O–complex 1	0.0	–	–	*C*_1_
H_2_O–complex 3	16.2	–	–	*C*_1_
(H_2_O)_2_–complex 2	0.0	–	–	*C*_1_
(H_2_O)_2_–complex 4	24.6^[a]^	–	–	*C*_1_

[a] Owing to unfavorable energy, frequencies not calculated.

To further judge whether these structures are likely to occur, we compare the dimerization energies to the energy gain as a result of decomposition into its components CO_2_ and methanol. For the decomposition of the CAME dimers 1–3, we obtain zero‐point corrected energies between −11.7 kJ mol^−1^ for dimer 1, −26.1 kJ mol^−1^ for dimer 2, and −40.6 kJ mol^−1^ for dimer 3, compared with zero‐point corrected dimerization energies of −69.4 kJ mol^−1^ (dimer 1), −75.2 kJ mol^−1^, and −82.8 kJ mol^−1^ (dimer 3). In contrast for the CA dimer, an earlier study found that the dimerization has almost the same energy as its decomposition into CO_2_ and H_2_O considering zero‐point energy corrected values, which are both about 67 kJ mol^−1^.^20^ Excluding entropic contributions, the decomposition of CAME dimers is significantly less favorable than decomposition of the CA dimer.

Although dimer 3 is built from two CAME monomers in the less favorable structure III conformation (1–2 s‐*trans*, 2–4 s‐*cis*), its high abundance in the solid (see section 2.3) can be rationalized as follows: (i) the crystal field, that is, the environment in the solid, may affect the conformational preference and shift the relative stability towards structure III; (ii) conversion between dimer 1 and dimer 3 may occur by synchronous double proton transfer of the two protons involved in the dimer bonds or by rotation of both terminal groups. Calculation of rate constants for double proton transfer in CAME dimers, including the possibility of quantum tunneling, is beyond the scope of this work. However, the proton exchange in CAME dimers can be compared with CA dimers; for example, formic acid or benzoic acid dimers, which show calculated rate constants of *k*≈10^9^–10^10^ s^−1^ at 300 K and ≈10^5^ s^−1^ at 30 K.^21^ Concerted proton transfer of benzoic acid at room temperature has been determined to exhibit an activation energy of approximately 5.4 kJ mol^−1^, which is lowered to an apparent activation energy of approximately 0.8 kJ mol^−1^ at temperatures below 50 K owing to quantum tunneling. The barrier for the formic acid dimer is about 8 kJ mol^−1^ higher.^21^ These comparisons suggest that double proton exchange might play a role in the gas phase at about 210 K or in the matrix at approximately 10 K. However, tunneling splittings associated with this are not observed in the spectra, suggesting that double proton transfer is too slow at 10 K. Based on our computational studies and considerations, dimers 1–3 are likely to occur in the matrix, whereas all other species are thermodynamically not favored and unlikely to be formed. Therefore, only dimers 1–3 will be considered for the spectral assignment. Also, conformational tunneling for s‐*trans*/s‐*cis* rotamerization is too slow in CAME to be observed experimentally in the form of tunneling splittings.^22^

#### 2.1.3 Structure and stability of CAME–water complexes

The presence of water vapor in the atmosphere and water as an impurity in the solvents may cause contamination of the matrix with water itself and CAME–water complexes. We studied several conformations for each of the two low‐energy conformations of the CAME monomer with one or two additional water molecules in various positions. Four CAME–water complexes with the following composition were found to be stable and constitute energy minima:

complex1=monomerI+1watermolecule

complex2=monomerI+2watermolecules

complex3=monomerII+1watermolecule

complex4=monomerII+2watermolecules

The structure of these complexes is displayed in Figure 2, whereas the relative electronic energies are listed in Table 1. Complex 4 was discarded for further analysis owing to its high relative energy. Other water complexes, for example, with structure III seem unlikely to occur owing to unfavorable stability. Water complexes 1 and 3 consist of structures I and II, where one water molecule forms two hydrogen bonds, one to the hydroxyl group and one to the carbonyl oxygen atom, giving rise to a distorted cyclic arrangement. In water complex 2 and complex 4, two water molecules form a cyclic structure with hydrogen bonds to the hydroxyl and the carbonyl O. For each water molecule, the oxygen and one hydrogen atom participate in the hydrogen‐bond network, whereas the other H atom points outwards. Notably, the water molecules are not in plane with the mirror plane of CAME but are out of plane.

#### 2.1.4 Calculated IR spectra

Infrared spectra of the most abundant monomers (structures I to III) and dimers (dimers 1 to 3) were calculated in the gas phase by employing ab initio wave function methods (MP2/ aug‐cc‐pVTZ). All frequencies obtained within the harmonic approximation were scaled by 0.98 as this ensures the least average deviation between experiment and calculation at wavenumbers below 2000 cm^−1^ (see tables in the following sections). The resulting deviation of theory and matrix isolation spectroscopy experiment below 2000 cm^−1^ is 4–8 cm^−1^ for the monomers and 13–22 and 25 cm^−1^ for dimers and water complexes. In general, the deviation between calculated and experimental frequencies above 2000 cm^−1^, especially for X‐H modes (X=C, O) is higher, in the present case 50–150 cm^−1^, because of pronounced anharmonicities and strong normal mode coupling.^23^

Calculated frequencies are plotted together with the experimental data as line spectra (see figures in the following sections). To aid the assignment, spectra of isotopically labeled CAME species complete our analyses. They comprise ^13^C‐CAME, CD_3_‐CAME, and OD‐CAME. For the spectral assignment, dimers 1, 2, and 3 were also calculated as isotopically labeled molecules for CH_3_/CD_3_, OD/OH, and ^13^C/^12^C substitution in the CAME molecule. As will be discussed in detail in the following chapter, the OD‐CAME and ^13^C‐CAME experiments show impurities of unlabeled CAME. Thus, isotopically mixed dimers need to be considered as well (OD‐OH dimers and ^13^C‐^12^C dimers, no impurities are found in the CD_3_‐CAME spectrum). This results in additional calculated spectroscopic data for one mixed dimer 1, two mixed dimers 2, and one mixed dimer 3 (for details see Quantum chemical setup in the Experimental Section). In the following matrix isolation spectroscopy figures, the intensities of all calculated dimer modes are displayed with one‐tenth of the initially calculated intensity, which is required for an appropriate match with the experimental data. For isotopically mixed dimers, the intensities of the calculated normal modes are scaled by 1/20. Finally, IR spectra of three CAME–water complexes were calculated. However, no isotopically labeled water complexes are shown in this work, as this would go beyond the scope of the discussion. The peak intensities of these complexes are displayed with one‐tenth of the initially calculated intensity (see Figure 6), which results in an appropriate match with the experimental data.

### 2.2 Experiments on the pure solid state: Variation of the solvents

The preparation of CAME under cryo‐conditions as a pure solid and subsequent matrix isolation was briefly described previously in reference 12, but here we want to provide a short discussion of the reaction pathway and the stability of the isolated product. In the present work, we divide the preparation into two steps: step (1) esterification of KHCO_3_, formation of the hemiester salt K[O_2_COCH_3_] in solvent 1 and step (2) protonation to CAME in solvent 2. This is illustrated and discussed in detail in the Supporting Information in Figure S1 ‘reaction pathway′ and the corresponding FTIR spectra of solid K[O_2_COCH_3_]/HO_2_COCH_3_ in Figure S2. Table S1 (in the Supporting Information) lists the observed IR frequencies of the hemiester and its potassium salt, providing a comparison of the K[O_2_COCH_3_] spectrum with the spectrum in the work of Behrendt et al.^24^ and a reinterpretation of the modes of solid CAME compared with the former ′alpha‐carbonic acid′ assignment of HHM.10b The newly assigned modes of CAME are highlighted in red in Table S1 (in the Supporting Information) and Table 2. To underline our band assignment in Table S1, Figure S3 (in the Supporting Information) shows the spectrum of solid CAME compared with the MP2/aug‐cc‐pVTZ calculated gas phase vibrational bands. Strong coupling between molecules and the crystal field severely broadens and shifts all bands in the spectra of solid CAME. Still, plotting the calculated in vacuo spectra together with the experimental FTIR spectrum of solid CAME in Figure S3 b (comparison to calculated line spectra of CAME dimers, in the Supporting Information) strongly supports the reassignment provided in Table S1 (in the Supporting Information) and Table 2. We are aware of the fact that isolated dimers also do not account for the crystal field properly. However, the cyclic dimer motif is energetically favorable and the improved match of the dimer spectra with the solid‐state spectra of CAME suggests dimers as basic building blocks of the solid.

**Table 2:** Comparative assignment of the IR frequencies of solid CAME, solid β‐H_2_CO_3_, and solid CAEE (all values in cm^−1^). Indicated in red are newly assigned bands of CAME.^[a]^

CAME		β‐H_2_CO_3_		CAEE	
expt. Figure 3 a	assignment Table S1	expt. Figure 3 b	assignment HHM10c	expt. Figure 3 c	assignment Bernard et al.^15^
3626	ν(OH)				
3510					
≈3340					
≈3250					
3153					
		3034	ν(C=O)+2×δ_ip_(CO_3_)	2994	ν(CH)
2990	ν(CH_3_)			2909	ν(CH) or 2×ν_as_[EtOCOH]
2918					
2880					
2766, 2747	ν(CH_3_), ν(OH)	2839	ν_as_[C(OH)_2_]+δ_ip_(COH) or 2×ν_as_[C(OH)_2_]+2×δ_ip_(CO_3_)	2723	ν_as_[EtOCOH]+δ_ip_(COH) or 2×ν_as_[EtOCOH]+2×δ_ip_(CO_3_)
				2652	
2700	ν(OH)	2619	2×δ_ip_(COH)	2569	2×δ_ip_(COH)
1786	ν(C=O)	1701	ν(C=O)	1730	ν(C=O)
1709					
1479	ν(C‐OH)	1503	ν_as_[C(OH)_2_]	1487	ν_as_[EtOCOH]
1464	δ_ip_(OH), δ_ip_(CO_3_), δ(CH_3_)			1466	δ(CH)
1447					
1423					
1325	δ_ip_(OH), δ_ip_(CO_3_), δ(CH_3_)	1298	δ_ip_(COH)	1379	δ_ip_(COH)
1312					
1250	ν(C‐OH)				
1200	δ(CH_3_)			1310	δ_s_(CH_3_)
1163				1163, 1121	ν(CO)
1086	ν(O‐CH_3_)	1034	ν_s_[C(OH)_2_]	1082	ν_s_[EtOCOH]
				1009	ν(CC)
912	δ_ip_(C‐O‐CH_3_)				
891	δ_oop_(OH)	876	δ_oop_(COH)	928, 901	δ_oop_(COH)
802	δ_oop_(CO_3_)	812	δ_oop_(CO_3_)	800	δ_oop_(CO_3_)
779					
702	δ_ip_(CO_3_)	683	δ_ip_(CO_3_)	583	δ_ip_(CO_3_)
660					
584		658			

[a] ν_s_ and ν_as_: symmetric and asymmetric stretching modes; δ_ip_ and δ_oop_: in‐plane and out‐of‐plane bending modes; δ_s_ and δ_as_: symmetric and asymmetric bending modes.

Targeted variation of solvents during preparation in steps (1) and (2) clearly illustrates the reaction pathway and the stability of the monomethyl ester of carbonic acid.

#### 2.2.1 Variation of solvents in step (1)

**Figure 3:**
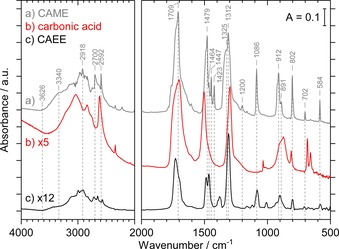
a) FTIR spectrum of CAME (HO_2_COCH_3_) after protonation of solid K[O_2_COCH_3_] with HCl in H_2_O, recorded at 80 K. b) FTIR spectrum of β**‐**H_2_CO_3_ after protonation of KHCO_3_ with HCl in H_2_O, recorded at 80 K. c) FTIR spectrum of CAEE (HO_2_COCH_2_CH_3_) after protonation of K[O_2_COCH_2_CH_3_] with HCl in H_2_O, recorded at 210 K. All spectra are taken from the solid in vacuo. Dashed lines mark characteristic bands of CAME.

Using different solvents for the dissolution of KHCO_3_ with the same experimental procedure, that is, by using water, methanol, or ethanol as the solvent for step (1) and subsequent uniform protonation with HCl in water in step (2), leads to the formation of β‐H_2_CO_3_, CAME, and CAEE,^15^ respectively. Acid‐catalyzed hydrolysis, however, does not take place under cryo‐conditions as shown previously in detail for carbonic acid ethyl ester (CAEE).^15^ Figure 3 provides a comparison of the spectra of solid CAME with solid β‐H_2_CO_3_ and CAEE after the exact same preparation procedure for all three solids with the only exception of varying the solvent in preparation step (1). Table 2 lists the bands of all three species including their vibrational assignment. This direct comparison demonstrates that the formation and isolation of the hemiesters (CAME and CAEE) is successful with no hydrolysis to H_2_CO_3_ occurring.

A clear distinction of the spectra of KHCO_3_ (by using water in the first step) and K[O_2_COCH_3_] (by using methanol in the first step) is possible, which is supported by comparison with the work of Nakamoto et al.^25^ (see Figure S4 and Table S2 in the Supporting Information). Note that detailed discussions of β‐H_2_CO_3_ and CAEE, including also matrix isolation, can be found in our earlier work.^12^, ^15^, ^19^

#### 2.2.2 Variation of solvents in step (2)

**Figure 4:**
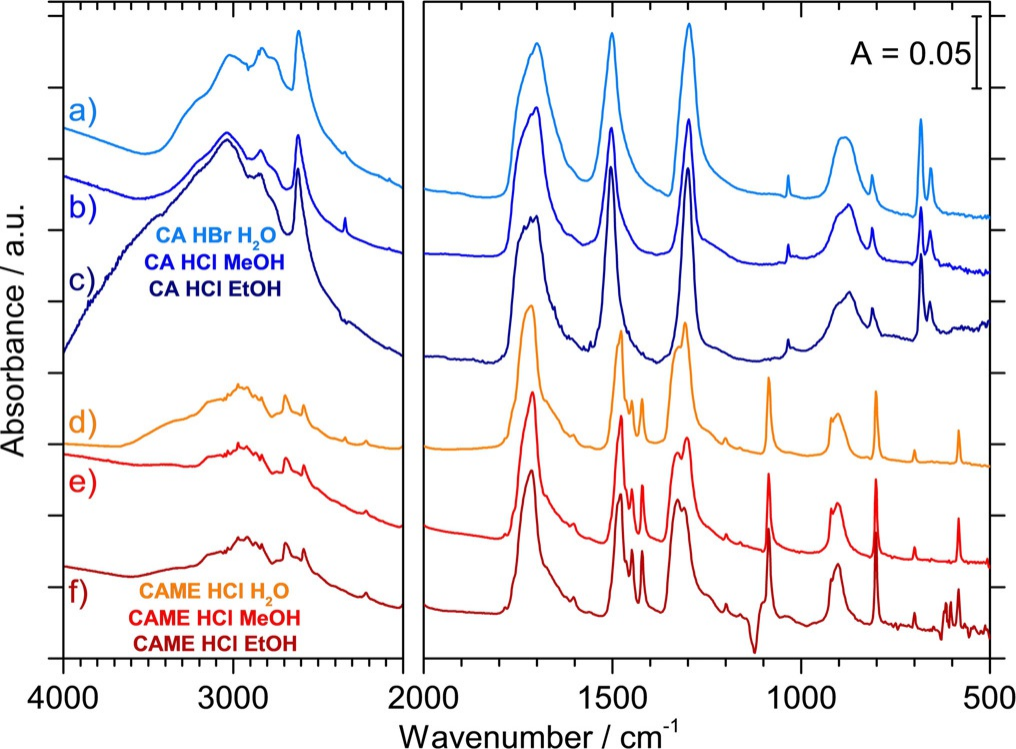
a)**–**c) Spectra of β**‐**carbonic acid (CA, β**‐**H_2_CO_3_) by protonation of KHCO_3_ with a) HBr in H_2_O (recorded at *T*=230 K), b) HCl in CH_3_OH (*T*=230 K), and c) HCl in C_2_H_5_OH (*T*=220 K). d)**–**f) Spectra of CAME by protonation of K[O_2_COCH_3_] with d) HCl in H_2_O (*T*=200 K), e) HCl in CH_3_OH (*T*=210 K), and f) HCl in C_2_H_5_OH (*T*=200 K).

Although variation of the solvent in step (1) has an impact on the reaction product, the variation of the solvent in step (2) has no impact. In Figure 4, spectra of β‐H_2_CO_3_ and CAME are shown, which were recorded after acidification and solvent evaporation. No matter which acidic solution (aqueous HBr, methanolic HCl, or ethanolic HCl) was used in step (2), the protonation of KHCO_3_ leads to the same type of spectrum originating from β‐H_2_CO_3_ (Figure 4 a–c).

Similarly, protonation of K[O_2_COCH_3_] leads to CAME regardless of whether aqueous, methanolic, or ethanolic HCl is used as the solvent. All spectra in Figure 4 d–f correspond to the CAME spectrum shown in Figure S2 b (in the Supporting Information).

### 2.3 Experiments on the pure solid state: Isotope labeling

To distinguish unequivocally between the interpretation as α‐H_2_CO_3_ and CAME, isotopically labeled solvents were used in both preparation steps as well as isotopically labeled KHCO_3_ to produce HO_2_COCD_3_, DO_2_COCH_3_, and HO_2_^13^COCH_3_. A precise assignment of all isotopically labeled CAME molecules is shown for the matrix isolation spectra, but for the FTIR spectra of the solid, CH_3_/CD_3_ exchange is presented as an example, where CD_3_OH was used as the solvent for KHCO_3_ instead of CH_3_OH.

**Figure 5:**
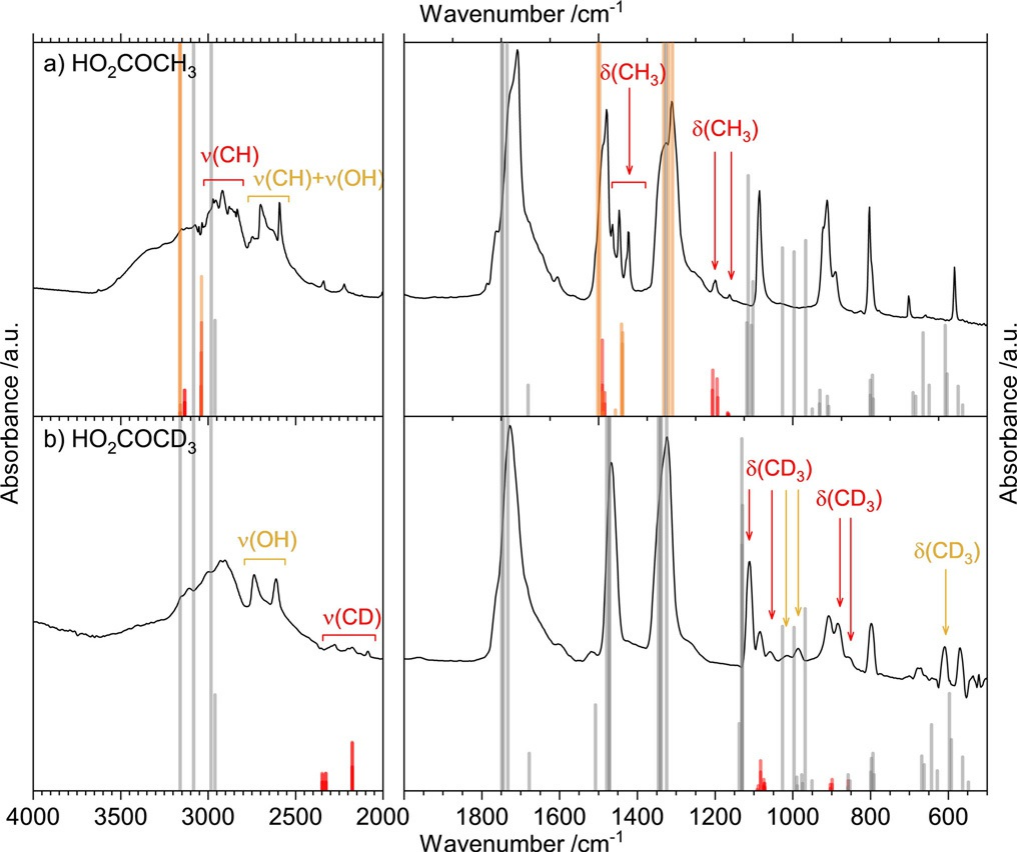
a) FTIR spectrum of solid HO_2_COCH_3_ recorded at 80 K. b) FTIR spectrum of solid HO_2_COCD_3_ recorded at 80 K. Calculated line spectra of HO_2_COCH_3_/HO_2_COCD_3_ dimers are labeled by the following color code: red=pure CH_3_/CD_3_ vibrations, gray=non**‐**CH_3_ vibrational modes/vibrational modes that are not coupled to CH_3_/CD_3_ vibrations, orange=vibrational modes coupled with CH_3_/CD_3_ vibrations.

A discussion of the spectral shifts of the solid precursor salts of CAME and CD_3_‐CAME is provided in the Supporting Information together with Figure S5. Figure 5 shows the spectra of solid CAME (a) and CD_3_‐CAME (b) together with the calculated line spectra of the dimers. The color code in Figure 5 and Table 3 is used to visualize the peak shift/splitting upon CH_3_/CD_3_ exchange: modes that are pure CH_3_/CD_3_ modes are labeled in red, bands that involve CH_3_/CD_3_ modes coupled to other modes are labeled in orange, and gray indicates modes that are not affected by isotopic labeling. Table 3 shows a comparison of the frequencies and its isotopically labeled analogue including the H/D ratio, perfectly matching the assignment and the shifts predicted by calculated modes. In general, substitution of a deuterium atom for a hydrogen atom redshifts the pure stretching modes by a factor of approximately √2. The bands related to the CH_3_ group that are expected to shift between 1.3 and 1.4 do indeed shift, namely ν(CH)/ν(CD) from 2990, 2918, and 2880 to 2276 and 2176 cm^−1^ and δ(CH_3_)/δ(CD_3_) shifts from 1200 and 1163 to 883 and 858 cm^−1^. Coupled modes that split are the ν(CH_3_)+ν(OH) mode from 2766/2747 into ν(CH_3_) at 2760 and ν(OH) at 2087, δ_ip_(OH)+δ_ip_(CO_3_)+δ(CH_3_) at 1464/1447/1423/1325/1312 to 1468/1323 and 1111/1056/1016/986 (for details, see Table 3). A mode at 610 cm^−1^, which appears for CD_3_‐CAME, can be assigned as a δ(CD_3_) mode. Typical bands that are unaffected by isotopic labeling are, for example, ν(OH), ν(C=O), or δ_oop_(CO_3_).

**Table 3:** Assignment of the IR frequencies of solid K[O_2_COCH_3_]/K[O_2_COCD_3_] and solid HO_2_COCH_3_/HO_2_COCD_3_ (all values in cm^−1^).^[a]^

K[O_2_COCH_3_]	K[O_2_COCD_3_]			HO_2_COCH_3_	HO_2_COCD_3_		
expt., Figure S5 a	expt., Figure S5 b	H/D ratio	assign.^24^	expt. Figure 5 a	expt. Figure 5 b	H/D ratio	assign.10b
				3626	≈3150	1.00	ν(OH)
				3510			
				≈3340			
				≈3250			
				3153			
2976	2245	1.33	ν_as_(CH)/ν_as_(CD)	2990	2276	1.31	ν(CH)/ν(CD)
				2918			
2949	2127	1.39		2880	2176	1.34	
2845	2077	1.37	ν_s_(CH)/ν_s_(CD)	2766, 2747	≈2760	1.00	ν(CH_3_), ν(OH)/ν(OH)
					2087	1.33	ν(CD)
				2700	2737	0.99	ν(OH)
					2612	1.03	
				≈2675			ν(CH_3_), ν(OH)
				≈2630			
				≈2675			ν(CH_3_), ν(OH)
				≈2630			
				2592			ν(OH)
				1786			ν(C=O)
1661	1666	1.00	ν(C=O)	1709	1728	0.99	ν(C=O)
				1607			ν(C=O)
				1479	1468	1.01	ν(C‐OH)/ δ_ip_(OH), δ_ip_(CO_3_)
1441	1107	1.30	δ(CH_3_)/δ(CD_3_)	1464	1111	1.32	δ_ip_(OH), δ_ip_(CO_3_), δ(CH_3_)
				1447			
				1423	1056	1.35	δ(CD_3_)
1310	1337	0.98	ν(C‐OC)+δ(CH_3_)/ν(C‐OC)	1325	1323	1.00	δ_ip_(OH), δ_ip_(CO_3_), δ(CH_3_)/δ_ip_(OH), δ_ip_(CO_3_)
	986	1.52	δ_ip_(C‐O‐CD_3_)	1312	1016, 986	1.33	δ_ip_(C‐O‐CD_3_)
				≈1250	≈1260	0.99	ν(C‐OH)
				1200	883	1.36	δ(CH_3_)/δ(CD_3_)
1186	860	1.38	δ(CH_3_)/δ(CD_3_)	1163	858		
1080	1069	1.01	ν(C‐O)	1086	1084	1.00	ν(O‐CH_3_)
901	903	1.00	ν(CH_3_O)	912	908	1.00	δ_ip_(C‐O‐CH_3_)
				891			δ_oop_(OH)
826	826	1.00	δ_oop_(CO_3_)	802	799	1.00	δ_oop_(CO_3_)
				779			
				702	679	1.03	δ_ip_(CO_3_)
				660			
683	513	1.33	δ_as_(CO_2_)				
			δ(CD_3_)				
					610		δ(CD_3_)
592	581	1.02	δ_s_(CO_2_)	584	569	1.03	δ_ip_(CO_3_)

[a] ν_s_ and ν_as_: symmetric and asymmetric stretching modes; δ_ip_ and δ_oop_: in‐plane and out‐of‐plane bending modes; δ_s_ and δ_as_: symmetric and asymmetric bending modes. Pure CH_3_ vibrational modes are labeled in red and modes that are decoupled upon isotopic labeling are labeled in orange. Modes that are not affected by CH_3_/CD_3_ exchange are labeled in gray.

These band shifts/splittings induced by using d_3_‐MeOH instead of MeOH clearly demonstrate the presence of the methyl group in the product and its origin from the solvent.

Note that according to the criteria established by Winkel et al.,^11^ CD_3_‐CAME is rather amorphous whereas CAME is mainly crystalline. This can also be recognized by comparing the FWHM of the bands. Thus, strictly speaking the H/D ratios listed in Table 3 not only include the shifts induced by the isotopic labeling (CH_3_ to CD_3_), but also small shifts related to the crystallization, which only took place for CAME (Figure 5 a), but not for CD_3_‐CAME (Figure 5 b).

The new assignment of solid‐state spectra supported by calculated line spectra and isotopic labeling in this work rules out the interpretation of the spectrum in Figure S2 b (in the Supporting Information) on the basis of α‐H_2_CO_3_. The high degree of similarity of the spectrum in Figure S2 b (in the Supporting Information) and the spectrum reported by HHM in their Figure 7 in ref. 10b suggests that their interpretation on the basis of α‐H_2_CO_3_ is incorrect. In particular, the presence of the bands assigned as CH modes in Table S1 (most notably bands at 1447 cm^−1^ and 1200 cm^−1^, in the Supporting Information) clearly speaks in favor of their product being CAME as well. This suggests that the sandwich technique, skipping the first evaporation of the solvent, used by HHM also involves K[O_2_COCH_3_] as an intermediate in solution. The fact that all modes pertaining to the methyl group are of low intensity explains why HHM had overlooked its presence and rather considered the presence of disordered carbonic acid or impurities as the origin for these weak bands.

### 2.4 Matrix isolation: Trapping in argon

Solid CAME is evaporated at 210 K in the matrix isolation setup, and the molecules above the solid are trapped in an Ar matrix at 10 K. The following figures show the results for CAME as well as its isotopically labeled isotopologues: CD_3_‐CAME, OD‐CAME, and ^13^C‐CAME. In addition, difference spectra before and after UV irradiation of the molecules trapped in the matrix below 10 K are shown to corroborate the assignment in section 2.5. The assignment of the experimental spectra is supported by MP2/aug‐cc‐pVTZ calculated line spectra, displayed together with the matrix isolation spectra, where a scaling factor of 0.98 was used. A full assignment is possible by considering not only monomer structures but also dimers and water complexes (see also the discussion in section 2.1).

#### 2.4.1 CAME—monomers, dimers, and water complexes

**Figure 6:**
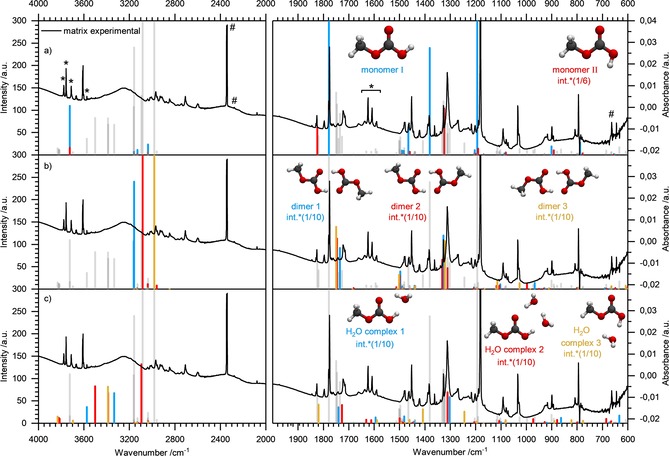
Matrix isolation spectra and MP2/aug**‐**cc**‐**pVTZ calculated spectra of CAME. a) Monomer I=blue, monomer II=red, dimers and water complexes =gray. b) Dimer 1=blue, dimer 2=red, dimer 3=orange, monomers and water complexes=gray. c) Water complex 1=blue, water complex 2=red, water complex 3=orange, monomers and dimers=gray. For the calculated line spectra, a 6:1 mixture of monomer I and II was assumed. The intensity of calculated lines of dimers and water complexes are divided by 10. Bands corresponding to CO_2_ and H_2_O are labeled with * and #. Calculated frequencies are scaled by a factor of 0.98.

Figure 6 shows the matrix spectrum of CAME in the region of 4000–600 cm^−1^. Apart from CAME monomers and dimers, other species identified in the spectrum are H_2_O and CO_2_. These are labeled with * and # in Figure 6 a. They may either be products of CAME decomposition or enter the matrix through the transfer procedure and/or leaks in the chamber. The experiments using ^13^C substitution (see below) indicate that CO_2_ in fact originates from the decomposition pathway. In addition, a trace amount of methanol is identified as an impurity based on the observation of the ν(C‐O) mode at 1034/1029 cm^−1^ and very weak ν(C−H) modes at 2956, 2929, 2921, 2913, 2909, 2848, and 2055 (2×1034) cm^−1^. This assignment is verified by separate matrix isolation experiments with pure MeOH in Ar (not shown here) and by comparison with the literature.^26^

For a better overview, the assignment of the matrix isolation spectrum of CAME is presented in three steps. For this reason, Figure 6 contains three rows (a, b, and c), all of which show the same spectrum. The spectrum is directly compared with calculations of: a) line spectra of monomer structures I and II, b) dimer structures 1, 2, and 3 and c) water complexes 1, 2, and 3. In a) blue and red lines indicate the calculated bands for monomer structures I and II, respectively. Gray lines indicate the line spectra of CAME dimer species and water complexes as shown in rows b and c, respectively. By scaling the calculated intensities using a ratio of 6:1, a good match with the calculated spectra is achieved. Most notably, the pattern of the strong ν(C=O) mode and the very intense δ_ip_(OH) modes can only be reproduced if a 6:1 ratio is chosen. Table 4 lists the observed and calculated peaks assigned to the monomers. The characteristic bands of structure I are the ν(OH/OD) mode at 3611/3608 cm^−1^, the ν(C=O) mode at 1779/1776 cm^−1^, and δ_ip_(OH) at 1182 cm^−1^. Weaker, but clearly assignable bands are the δ_s_(CH_3_) modes at 1452 and 1445 cm^−1^, the δ_ip_(OH)+δ_ip_(CO_3_)+δ(CH_3_) mode at 1389/1383 cm^−1^, ν(C‐OCH_3_)+δ(CH_3_) modes at 1193 and 1189 cm^−1^, the δ_ip_(C‐O‐CH_3_) mode at 899 cm^−1^, and the δ_oop_(CO_3_) mode at 794 cm^−1^. The assignment of the stretching modes of the CH_3_ group of the ester is challenging owing to the overlap with the stretching modes of the OH and CH_3_ groups of the dimers and water complexes. However, considering the complete picture (see below), bands at 3041, 3005, and 2916 cm^−1^ can be assigned to ν_as_(CH_3_) and ν_s_(CH_3_). For weak bands that are superimposed with bands originating from dimers or water complexes, a clear assignment can be made based on the difference spectra after UV irradiation (see Figure 8 in section 2.5). For monomer structure II, the most obvious bands are the ν(OH), ν(C=O), and δ_ip_(OH)+δ_ip_(CO_3_) bands at 3602, 1830/1826, and 1327 cm^−1^. The δ_s_(CH_3_) band can be detected at 1445 cm^−1^. With the help of difference spectroscopy, very weak bands at 1072, 894, and 786 cm^−1^ can be clearly assigned to ν(O‐CH_3_)+ν(C‐OH), δ_ip_(C‐O‐CH_3_), and δ_oop_(CO_3_). These findings are in good agreement with the bands observed by Reisenauer et al., who isolated CAME through a high vacuum flash pyrolysis process.^16^ Whereas the assignment of monomer structure I and II is based on the observation of *all* significant bands in the calculation, the presence of structure III is indicated solely based on the most intense band at 1797 cm^−1^, corresponding to its ν(C=O) mode. This assignment is doubtful as other normal modes, for example, those arising from CAME–water complexes, might be at the origin of the band (see discussion below). In other words, either structure III is absent in the matrix or if the 1797 cm^−1^ band originates from it, then it is less abundant by a factor of at least 10.

**Table 4:** Assignment of IR frequencies of monomer I and II of CAME, CD_3_ **‐**CAME, OD**‐**CAME, and ^13^C**‐**CAME (all values in cm^−1^).^[a]^

CAME		CD_3_‐CAME		H/D shift		OD‐CAME		H/D shift		^13^C‐CAME		^12^C/^13^C shift		assign.	
Ar	calcd	Ar	calcd	Ar	calcd	Ar	calcd	Ar	calcd	Ar	calcd	Ar	calcd		
**3611/3608** ^[b,c]^	**3724**	3610	3724	1.00	1.00	2665/2663	2710	1.35	1.37	**3610**	**3724**	1.00	1.00	mono1	ν(OH/OD)
**3602** ^[b,c]^	**3725**					2660	2710	1.35	1.37	**3602**	**3725**	1.00	1.00	mono2	ν(OH/OD)
3041^[b,c]^	3160	2285	2347	1.33	1.35					3041	3160	1.00	1.00	mono1	ν_as_(CH_3_/CD_3_)
		2280													
3005^[b,c]^	3129	2266	2324	1.33	1.35					3005	3129	1.00	1.00	mono1	ν_as_(CH_3_/CD_3_)
		2244													
2916^[b,c]^	3037	2101	2174	1.39	1.40					2916	3037	1.00	1.00	mono1	ν_s_(CH_3_/CD_3_)
		2088													
**1830/1826** ^[b,c]^	**1824**	1826/1824	1822	1.00	1.00	1822/1818	1817	1.00	1.00	**1790/1787**	**1778**	1.02	1.03	mono2	ν(C=O)
**1779/1776** ^[b,c]^	**1778**	1778/1774	1776	1.00	1.00	1774/1770	1772	1.00	1.00	**1735/1733**	**1733**	1.03	1.03	mono1	ν(C=O)
**1452** ^[b,c]^	**1465**					1449	1463	1.00	1.00	**1447**	**1460**	1.00	1.00	mono1	δ_s_(CH_3_)
**1445** ^[b,c]^	**1459**									**1442**	**1457**	1.00	1.00	mono2	δ_s_(CH_3_)
		1401	1397											mono1	δ_ip_(OH), δ_ip_(CO_3_)
**1389/1383** ^[b,c]^	**1381**	988	983	1.40	1.40					**1362**	**1354**	1.02	1.02	mono1	δ_ip_(OH), δ_ip_(CO_3_), δ(CH_3_)
**1327**	**1324**	1337	1336	0.99	0.99					**1309**	**1304**	1.02	1.02	mono2	δ_ip_(OH), δ_ip_(CO_3_)
						1329 sh	1337	1.05	1.03					mono1	δ_ip_(CO_3_)
						1268	1284							mono2	δ_ip_(CO_3_)
1193^[b,c]^	1204													mono1	ν(C‐OCH_3_), δ(CH_3_)
1189^[b]^															
**1182** ^[b,c]^	**1194**	1195/1188	1198	0.99	1.00					**1175/1174**	**1187**	1.01	1.01	mono1	δ_ip_(OH)
		1115	1126											mono1	ν(O‐CD_3_)
										**1170**	**1178**			mono2	δ_ip_(OH)
		1106	1119											mono2	ν(O‐CD_3_)
						1080/1079	1089							mono1	ν(O‐CH_3_), ν(C‐OD)
**1072** ^[b,c]^	**1081**									**1070**	**1079**	1.00	1.00	mono2	ν(O‐CH_3_), ν(C‐OH)
						1016/1014	1010							mono1	δ_ip_(OD), ν(C‐OD)
		905	905											mono1	δ(CD_3_)
**899** ^[b,c]^	**901**	832	832	1.08	1.08					**896**	**898**	1.00	1.00	mono1	δ_ip_(C‐O‐CH_3_), δ_ip_(C‐O‐CD_3_)
**894** ^[b]^	**892**									**890**	**888**	1.00	1.00	mono2	δ_ip_(C‐O‐CH_3_)
						865	862							mono2	δ_ip_(OD), δ_ip_(C‐O‐CH_3_)
						850	848							mono1	δ_ip_(OD), δ_ip_(C‐O‐CH_3_)
**794** ^[b,c]^	**788**	792	785	1.00	1.00	794	788	1.00	1.00	**770**	**764**	1.03	1.03	mono1	δ_oop_(CO_3_)
**786** ^[b]^	**778**					786	778	1.00	1.00	**762**	**754**	1.03	1.03	mono2	δ_oop_(CO_3_)
X̄${}_{\mathrm{th}\text{-}\exp\;>2000\;\mathrm{cm}{}^{-1}}$ mono1	119.5		77.1				46.0				119.5				
X̄${}_{\mathrm{th}\text{-}\exp\;<2000\;\mathrm{cm}{}^{-1}}$ mono1	7.9		4.3				6.3				7.0				
X̄${}_{\mathrm{th}\text{-}\exp\;>2000\;\mathrm{cm}{}^{-1}}$ mono2	123.0		‐				50.0				123.0				
X̄${}_{\mathrm{th}\text{-}\exp\;<2000\;\mathrm{cm}{}^{-1}}$ mono2	6.7		5.7				7.5				8.1

[a] Bold=validated by UV. Factor calc.=0.98. ν=stretching mode, δ=bending mode, index s=symmetric, as=asymmetric, ip=in plane, oop=out of plane, sh=shoulder. Typical isotopic shifts are labeled in red. X̄${}_{\mathrm{th}\text{-}\exp\;>2000\;\mathrm{cm}{}^{-1}}$ =average deviation theory–experiment >2000 cm^−1^; X̄${}_{\mathrm{th}\text{-}\exp\;<2000\;\mathrm{cm}{}^{-1}}$ =average deviation theory–experiment <2000 cm^−1^. [b] CAME impurity in OD‐CAME. [c] CAME impurity in ^13^C‐CAME.

Bands that are unexplained by CAME monomer conformers are compared with CAME dimer bands in Figure 6 b. Line spectra of three possible dimers, 1, 2, and 3, in Figure 6 are displayed in color with the same intensity (a tenth of the calculated values). The presence of these dimers allows for explanation of the bands in the region between 3050 and 2550 cm^−1^ and broad bands at approximately 1720, 1480, 1310, and 1090 cm^−1^. A detailed assignment of all dimer signals is shown in Table 5. The most prominent dimer modes are the following: ν(OH) (+ν(CH_3_)) at 3017 (dimer 1), 3005 (1), 2929 (2), and 2829 (3) cm^−1^, ν(C=O) at 1722 (3), 1720 (2), and 1708 (1) cm^−1^, δ_ip_(OH)+δ_ip_(CO_3_)+δ(CH_3_) at 1486 (1, 2, and 3), δ_ip_(OH) and/or δ_ip_(OH)+ν(C‐OCH_3_) at 1312 (2, 1) cm^−1^, and ν(O‐CH_3_/O‐CD_3_) at 1092/1079 (1, 2, and 3) cm^−1^. The calculated OH stretching modes of all dimers are strongly shifted owing to the challenges accompanied with the calculation of hydrogen bonds (see also discussion in section 2.1.3). Overall, the ratio of dimers to monomers is about 1:9 based on the observed intensities. Assessing the fractions of dimers 1, 2, and 3 individually is not possible because the two most intense bands appear as a broad band rather than three well‐separated peaks.

**Table 5:** Assignment of IR frequencies of dimers 1, 2, and 3 of CAME, CD_3_ **‐**CAME, OD**‐**CAME, and ^13^C**‐**CAME (all values in cm^−1^).^[a]^

CAME		CD_3_‐CAME		H/D shift		OD‐CAME		H/D shift		^13^C‐CAME		^12^C/^13^C shift		assign.	
Ar	calcd	Ar	calcd	Ar	calcd	Ar	calcd	Ar	calcd	Ar	calcd	Ar	calcd	dimer	
3017^[b,c]^	3163									3017	3163	1.00	1.00	1	ν(OH/OD), ν(CH_3_)
3005^[c]^	3158									3005	3158	1.00	1.00	1	
2929^[b,c]^	3083					2229	2306	1.33	1.34	2930	3083	1.00	1.00	1	ν(OH/OD)
						2196	2251							2	
2829	2982					2126	2180	1.33	1.34					3	
1722^[b,c]^	1749	1760	1749	0.98	1.00	1717 broad	1743	1.00	1.00	1703	1704	1.01	1.03	3	ν(C=O)
1720^[b]^	1747	1758	1745	0.99	1.00		1739	1.00	1.00	1697	1702	1.01	1.03	2	
1708	1735	1743	1733	0.98	1.00		1729	0.99	1.00	1685, 1683/1681	1691	1.01	1.03	1	
1486	1502									1478	1494	1.01	1.01	3	δ_ip_(OH), δ_ip_(CO_3_), δ(CH_3_)
	1502									1430	1437	1.04	1.04	2	
	1497													1	
		1472	1502											1	δ_ip_(OH), δ_ip_(CO_3_)
		1460	1479											2	
										1465	1486			2	δ_ip_(CO_3_), δ_ip_(OH), δ(CH_3_)
										1460	1497			1	
										1430	1436			3	
										1430	1434			2	
1429^[b]^	1441													1	δ_ip_(OH), δ(CH_3_)
1423^[b]^	1438													2	
1421^[b]^	1439													3	
						1376	1394							2	δ_ip_(CO_3_), δ(CH_3_)
						1366	1381							1	
						1351	1360							2	
						1351	1360							3	
1312^[b]^ (broad)	1332	1321	1368	0.99	0.97									2	δ_ip_(OH)
1312^[b]^ (broad)	1327	1321	1341	0.99	0.99									1	δ_ip_(OH), ν(C‐OCH_3_/C‐OCD_3_)
1285	1320	1313	1339	0.98	0.99									3	δ_ip_(CO_3_), ν(C‐OCH_3_/C‐OCD_3_)
1276^[b]^	1310													2	δ_ip_(OH)
										broad 1299–1281	1312			2	δ_ip_(CO_3_), δ_ip_(OH), δ(CH_3_)
										max.=1286	1307			1	δ_ip_(CO_3_), δ_ip_(OH)
											1299			3	δ_ip_(CO_3_), δ_ip_(OH)
											1290			2	δ_ip_(CO_3_), δ_ip_(OH), δ(CH_3_)
1216^[b,c]^	1207					1204, 1200	1207	1.01	1.00					2	δ(CH_3_)
1202^[b,c]^	1206					1204, 1200	1206	1.01	1.00					1	δ(CH_3_), ν(C‐OCH_3_)
	1195					1186	1195	1.01	1.00					3	δ(CH_3_)
	1193					1186	1195	1.01	1.00					2	δ(CH_3_)
1092^[b,c]^	1118	1128	1131	0.97	0.99					1092	1114	0.99	1.00	2	ν(O‐CH_3_/O‐CD_3_)
1079^[b,c]^	1115	1128	1153	0.97	0.96					1078, 1072	1102	1.01	1.00	3	
	1007													2	
	1103													1	
						1075, 1065, 1042, 1023/1021	1101, 1073							1 2 3	ν(O‐CH_3_), δ_ip_(OH/OD)
							1125, 1106, 1093, 1061								
							1123, 1056								
		1007	997											2	δ_oop_(OH)
						882	893							3	ν(C‐OCH_3_)
		861	848											3	δ(CH_3_/CD_3_)
						813	805			783	776	1.04	1.04	1, 2	δ_oop_(CO_3_)
						810	801			783	770	1.03	1.04	3	
										772	768			2	
X̄${}_{\mathrm{th}\text{-}\exp\;>2000\;\mathrm{cm}{}^{-1}}$	151.5		–				62.0				150.7				
X̄${}_{\mathrm{th}\text{-}\exp\;<2000\;\mathrm{cm}{}^{-1}}$	21.9		18.9				14.2				13.2

[a] Factor calc.=0.98. ν=stretching mode, δ=bending mode, ip=in plane, oop=out of plane. Typical isotopic shifts are labeled in red. X̄${}_{\mathrm{th}\text{-}\exp\;>2000\;\mathrm{cm}{}^{-1}}$ =average deviation theory–experiment >2000 cm^−1^ of all dimers; X̄${}_{\mathrm{th}\text{-}\exp\;<2000\;\mathrm{cm}{}^{-1}}$ =average deviation theory–experiment <2000 cm^−1^of all dimers. [b] CAME impurity in OD‐CAME. [c] CAME impurity in ^13^C‐CAME.

Even after the assignment of modes to CAME monomers and dimers, several bands remain unexplained. Thus, CAME–water clusters are considered in Figure 6 c, namely two clusters containing one water molecule and one cluster containing two water molecules. Similar to the CAME dimers, the calculated OH stretching frequencies are also shifted to higher wavenumbers for the CAME–water complexes owing to intermolecular hydrogen bonding. The spectral region above 2550 cm^−1^ is hard to assign to individual clusters (the only band assigned here is the ν(OH) mode of complex 2 at 2956 cm^−1^), but without consideration of water complexes the large number of bands in this region would remain unexplained. Table S3 (in the Supporting Information) lists the assigned peaks, including a column with an assignment as to whether the CAME or the water molecule contribute to the respective vibrational mode. The most prominent bands explained by the presence of CAME–water complexes are the ν(C=O) modes at 1797 (complex 3), 1722 (1), and 1715 (2) cm^−1^, δ_ip_(CO_3_)+ δ_ip_(OH) at 1389 (3), 1276 (2), and 1272/1269 (1) cm^−1^, ν(O‐CH_3_) at 1092 (2) and 1079 (1, 3) cm^−1^, and δ_oop_(OH)/δ_oop_(O‐H‐OH_2_) at 924 (2), 920/917 (1), and 808 (3) cm^−1^.

#### 2.4.2 Isotopologues

**Figure 7:**
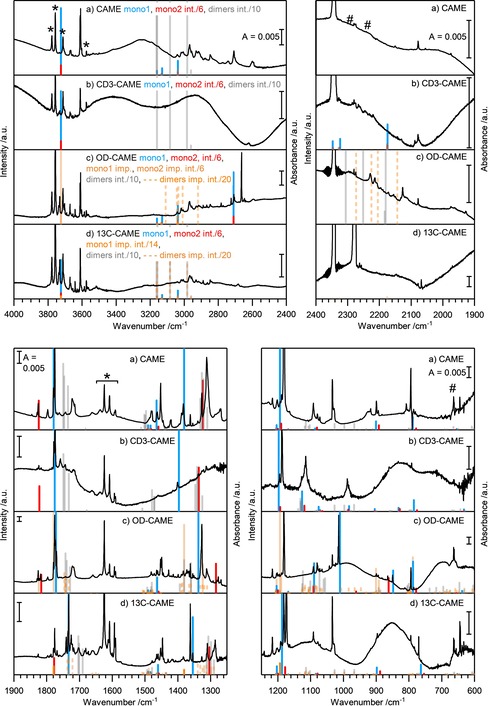
Matrix isolation spectra and MP2/aug**‐**cc**‐**pVTZ calculated spectra of a) CAME, b) CD_3_ **‐**CAME, c) OD**‐**CAME, and d) ^13^C**‐**CAME. For the calculated line spectra, a 6:1 mixture of structures I (blue) and II (red) was assumed. Isotopic monomer impurities were considered with a ratio of OD/OH=1:1 and ^13^C/^12^C=14:1 (^12^C**‐**CAME is labeled in orange in d)). Dimers and water complexes are labeled in gray and are scaled down to intensity/10. OD/OH impurity dimers in c) are labeled in orange with an intensity/20 and ^13^C/^12^C impurity dimers in d) are indicated by dashed gray lines with an intensity/20. Bands corresponding to CO_2_ and H_2_O are labeled with * and #. Calculated frequencies are scaled by a factor of 0.98.

CD_3_‐CAME (HO_2_COCD_3_) was prepared by using HO‐CD_3_ as the solvent in step (1) of the preparation. ^13^C‐CAME (HO_2_^13^COCH_3_) was prepared by dissolving KH^13^CO_3_ in step (1), and OD‐CAME (DO_2_COCH_3_) was generated by acidifying the salt in step (2) with DCl. Figure 7 separates the spectrum into four spectral ranges. Each spectral range consists of four panels: (a) pure CAME, (b) CD_3_‐CAME, (c) OD‐CAME, and (d) ^13^C‐CAME. The matrix isolation experiments of these labeled species (Figure 7) also show the same ratio of 6:1 of monomer structure I/II. The calculated line spectrum for structure I and structure II in this ratio is again indicated by blue and red lines in all panels in Figure 7. Again, the intensity of the dimers is a tenth—corroborating the 9:1 monomer/dimer ratio. Dimers are indicated by gray lines. The interpretation of the matrix spectra in Figure 7 reveals impurities of unlabeled CAME in the OD‐CAME and ^13^C‐CAME spectra. A ratio of 1:1 for OH/OD‐CAME and 1:14 for ^12^C/^13^C‐CAME is deduced from the intensity ratios of bands shifted upon substitution. Line spectra of the unlabeled monomer structures are included in Figure 7 c and d with the respective intensities. All bands found in the pure CAME spectrum as well as in the OD‐ and ^13^C‐CAME spectra as an impurity are labeled with * and # in Table 4, Table S3 (in the Supporting Information), and Table 5. Mixed OD‐OH and ^13^C‐^12^C dimers need to be considered as well. Energetically plausible dimers (same considerations as for all molecules shown, see section 2.1.1) are displayed with a twentieth of the calculated intensity. Possible mixed impurity dimers to be found in the matrix isolation spectrum are dimers 1 and 3 and two distinguishable dimer 2 complements. Other impurities from water, carbon dioxide, and methanol are labeled, but do not interfere with the hemiester bands. No bands of d_3_‐methanol (HO‐CD_3_) are found in the spectrum of CD_3_‐CAME in Figure 7 b and the only detectable peak of methanol in Figure 7 c and d is a signal at 1034/1029 cm^−1^, representing the ν(C‐O) mode.

All bands assigned to monomer I and II are listed in Table 4, including H/D shifts and ^12^C/^13^C shifts. Table 5 lists all bands of the dimer structures 1, 2, and 3, including isotopic shifts. Bands assigned to CH/CD and OH/OD modes shift with a typical factor of 1.33–1.40, whereas ^12^C/^13^C substitution shifts the bands by a factor 1.02–1.03. Other modes are coupled vibrations of isotopically labeled and unlabeled parts of the molecule and, thus, these signals “are split” or “disappear” and new peaks are observed, which cannot be associated with respective signals in the CAME spectrum (see also discussion in sections 2.3 and 2.6.2). It is nevertheless possible to assign these peaks thanks to the excellent accordance with the calculated spectra.

A detailed description and relevant statements that can be made about the spectra shown in Figure 7 are discussed in the Supporting Information. Tables S4 and S5 (in the Supporting Information) list all band assignment of mixed OD‐OH and ^13^C‐^12^C dimers.

To sum up this discussion of the isotopically labeled experiments, an excellent assignment of all spectra could be reached, which is in accordance with calculated spectra both in terms of band positions and isotopic shifts. From this interpretation, it is concluded that the cryo‐preparation solely leads to formation of carbonic acid monomethyl ester by the proposed mechanism in two steps. It is possible to assign almost all peaks of the full spectral range between 4000 and 600 cm^−1^ including dimers and water complexes of the hemiester. All shifts induced by isotopic labeling are plausible and match predictions from the calculated data. Impurities of unlabeled CAME in the OD‐ and ^13^C‐CAME experiments do not compromise the analysis, but rather consideration of mixed dimers confirms the actual peak assignment of the CAME matrix isolation spectra.

For all matrix isolation experiments in sections 2.4 and 2.5 discussed together with calculated line spectra considering monomers, dimers, and water complexes, a very comprehensive assignment of almost all signals is possible. A handful of peaks remain after this assignment, which are without exception of low intensity and are mainly found in the region above 2000 cm^−1^—the region of various OH and CH_3_ modes, especially of dimers and complexes. Complexes that were not considered are, for example, monomer+methanol, methanol+water, dimers including monomer II and water complexes with monomer structure III.

### 2.5 Matrix isolation: UV irradiation

**Figure 8:**
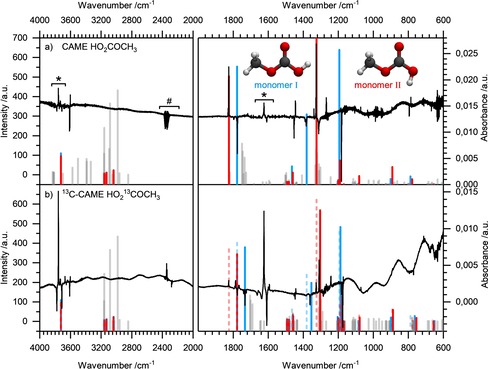
Difference spectra after matrix isolation prior to and after UV irradiation. MP2/aug**‐**cc**‐**pVTZ calculated line spectra of CAME and ^13^C**‐**CAME are included: a) CAME, monomer 1=blue, monomer 2=red, and dimers 1, 2, and 3=gray. b) ^13^C**‐**CAME, monomer 1=blue, monomer 2=red and ^13^C**‐**dimers 1, 2, and 3=gray; ^12^C impurities of monomer I and II are labeled with dashed lines. The calculated line spectra of monomers are shown with maximum intensity and lines of dimers are shown with intensity/10. Bands corresponding to CO_2_ and H_2_O are labeled with * and #. Calculated frequencies are scaled by a factor of 0.98.

UV irradiation and subsequent analysis by using difference spectra (between experiments prior to and after irradiation) was performed to aid the assignment, similar to the case of matrix isolated carbonic acid.^19^ UV irradiation causes isomerization, specifically from monomer structure I to structure II. In the present case, the energy transmitted by ultraviolet light induces the rotation of the C−OH bond by 180° with a barrier of 42.5 kJ mol^−1^ (see Figure 1 b). The barrier between monomer structure I and III is just slightly higher (45.5 kJ mol^−1^ for the rotation of the C−OCH_3_ bond by 180°) but structure III cannot be identified after UV irradiation. This might be because the minimum of monomer structure III is 8.8 kJ mol^−1^ higher than that for structure II and, thus, the back reaction to structure I has a lower barrier, making it too fast to observe structure III in the subsequent IR measurement. Furthermore, rotation of the C−OCH_3_ group in the argon cage might in fact have a higher barrier than the one indicated in Figure 1 b from in vacuo calculations. It is conceivable that rotation around the C−OH bond is easier within the cage than that around the C−OCH_3_ bond. Other than monomer isomerization, UV irradiation does not cause any additional changes—dimers and water complexes remain unaffected. That is, UV irradiation is ideally suitable to discriminate between the two monomer isomers and to identify bands that are not caused by either of the two monomer conformers. In the difference spectra in Figure 8, bands pertaining to structure II point upwards and bands pertaining to structure I point downwards. Bands of other species do not contribute to the difference spectra, that is, they show a difference of zero. UV irradiation also does not trigger decomposition of the molecules captured in the matrix to CO_2_, water, and MeOH.

Figure 8 shows the difference spectra of CAME (a) and ^13^C‐CAME (b) after 10 min UV irradiation. Bands of monomer structure I and II that are identified by these additional experiments are printed in bold in Table 4. Bands pointing downward pertaining to monomer I of CAME (Figure 8 a) are 3612/3609, 1779/1776, 1452, 1389/1383, 1182, 899, and 794 cm^−1^. That is, all modes assigned previously to structure I appear in the difference spectra, except for the ν(CH_3_) and ν(C‐OCH_3_)+δ(CH_3_) modes, which are too weak to cause a signal exceeding the noise in the difference spectra. Bands pointing upward pertaining to monomer II of CAME (Figure 8 a) are 3602, 1829/1826, 1445, 1326, 1072, 894, and 785 cm^−1^, in agreement with the previous assignment based on calculations.

In Figure 8 b, ^13^C‐CAME signals of monomer I pointing downward are observed at 3610, 1735/1733, 1447, 1362, 1175, 896, and 770 cm^−1^. Signals of monomer II pointing upward are detected at 1780/1776, 1452, 1182, and 794 cm^−1^. In addition to the ^13^C species, also ^12^C monomers appear in the difference spectrum: monomer I (downward bands) is detected at 1830/1826, 1445, and 1327 cm^−1^, and monomer II (upward bands) induces signals at 3602, 1791/1787, 1443, 1309, 1172, 1070, 890, and 762 cm^−1^. With the exception of the weak ν(CH_3_) modes, all signals assigned to monomers of ^13^C‐CAME in the previous experiments are confirmed after UV irradiation. Band positions in the UV irradiation experiments match excellently (difference less than 1 cm^−1^) with the ones assigned in Table 4—only for a couple of bands there is a shift of 1–2 cm^−1^. An unexplained weak band that appears in the UV irradiation spectra is the band pointing downward at 1797 cm^−1^. This suggests that it might arise from structure III rearranging to structure I. However, no other bands pertaining to structure III can be identified. In addition, there are two weak bands pointing upward (1268 and 1339 cm^−1^) and one band pointing downward (1312 cm^−1^), which cannot be explained based on conformational changes. In Figure 8 b, only the band at 1242 cm^−1^ remains unexplained.

### 2.6 Matrix isolation: Interpretation and discussion

#### 2.6.1 Comparison with CA and CAEE

Figure S6 (in the Supporting Information) shows a direct comparison of the FTIR spectra of matrix isolation experiments of CAME (a), carbonic acid (CA, b),^19^ and carbonic acid monoethyl ester (CAEE, c).^15^ All three spectra are based on an identical preparation as described in the Experimental Section. The only difference is the choice of the solvent used in step (1) of the preparation: a) methanol, b) water, and c) ethanol. No evidence of non‐esterified carbonic acid, which is referred to as β‐H_2_CO_3_ in the literature,10c, 19 is detected in the FTIR spectra of the solid and in the matrix isolation experiment.

In Figure S6 (in the Supporting Information), impurities of water and carbon dioxide are marked in red. The OH stretching mode, which appears at nearly the same wavenumbers for all three species, is colored in blue. The apparent OH and CH vibrations of CAME and CAEE in the spectral region above 2550 cm^−1^ are highlighted in orange. Most importantly, two distinct bands (ν(C=O) and δ_ip_(C‐OH)) of carbonic acid are marked in green. It is clear that absolutely no signs of these bands at 1792/1789 and 1136 cm^−1^ are observed in the spectra of CAME and CAEE. That is, the original assignment given in reference 12 on the basis of carbonic acid needs to be corrected, which is done in Table 6. Reassignments are labeled in red. The modes in reference 12 concerning the two OH groups in carbonic acid need to be reassigned as the monomethyl ester of carbonic acid provides one OH and an O‐CH_3_ group. This includes the ν_s/as_(OH) and ν_as_(C(OH)_2_) modes, whereas the latter is a δ_s_(CH_3_) vibration of the ester. Signals around 1270 cm^−1^ (CAME) with a former assignment as δ_ip_(COH) belong to water complex 1 and bands at 808 (CAME) and 784 (^13^C‐CAME) cm^−1^ are reassigned to δ_oop_(OH) of water complex 3 and δ_oop_(CO_3_) of dimers 1, 2, and 3. This rectification is complemented by the very comprehensive assignment of nearly all other bands of the whole FTIR spectra of CAME and its isotopically labeled equivalents, including dimers and water complexes. By comparing CAME and CAEE, we can conclude that also for CAEE some dimers can be trapped in the Ar matrix, for example, broad peaks of ν(C=O) or δ_ip_(OH) modes around 1720 and 1310 cm^−1^ but dimer bands are much more dominant in the CAME spectrum than in the CAEE spectrum. The correct matrix bands of carbonic acid after sublimation of solid β‐CA are given in reference 19.

**Table 6:** Rectification of the matrix isolation band assignment in Bernard et al., 2011.^[a][12]^

CAME		OD‐CAME		^13^C‐CAME		sc reference^12^		new assign.	
Ar^12^	Ar	Ar^12^	Ar	Ar^12^	Ar				
3611	3611/3608	2665	2665/2663	3610	3610	mono 1, 2	ν_s_(OH)	mono 1	ν(OH/OD)
3608		2663		3607		mono 1	ν_as_(OH)		
3604	3602	2660	2660	3603	3602	mono 2	ν_as_(OH)	mono 2	ν(OH/OD)
3602		2660		3601		mono 1	ν(OH)		
1829/1826	1830/1826	1822/1819	1822/1818	1791/1787	1790/1787	mono 2	ν(C=O)	mono 2	ν(C=O)
1779/1797	1779/1776	1774/1770	1774/1770	1768/1740	1735/1733	mono 1	ν(C=O)	mono 1	ν(C=O)
1722/1714									
1779/1776				1735/1733					
1452/1451	1452		1449		1447	mono 1	ν_as_(C(OH)_2_)	mono 1	δ_s_(CH_3_)
≈1270	1272/1269					mono 1	δ_ip_(COH)	H_2_O complex 1	δ_ip_(CO_3_), δ_ip_(OH)
≈1175						mono 2	δ_ip_(COH)	–	–
1182/1181	1182	1016		1175/1174	1175/1174	mono 1	δ_ip_(COH)	mono 1	δ_ip_(OH)
808	808			784	783	mono 1	δ_oop_(CO_3_)	H_2_O complex 3	δ_oop_(OH)
								dimer 1, 2, 3	δ_oop_(CO_3_)
794	794	794	974	772/770	770	mono 1	δ_oop_(CO_3_)	mono 1	δ_oop_(CO_3_)
785	786	785	786	762	762	mono 2	δ_oop_(CO_3_)	mono 2	δ_oop_(CO_3_)

[a] Labeled in red: reassigned bands.

#### 2.6.2 CH_3_ modes of CAME—A retrospective discussion

Similar to the discussion of the FTIR spectra of solid CAME in Figure 5, Figure S7 (in the Supporting Information) also shows an alternative representation of calculated line spectra to demonstrate the shifts induced by CH_3_/CD_3_ substitution. The calculated line spectra shown include monomer structure I and II (int/6) and dimer structures 1, 2, and 3 (int/10) analogous to Figure 6 but using a different color scheme: all peaks that do not exhibit a CH_3_/CD_3_ mode are gray, for example, ν(OH) or ν(C=O) at 3724 and 1778 cm^−1^ (monomer I, wavenumber calculated×0.98), δ(CH_3_) or ν(CH_3_) vibrations are labeled in red, for example, around 3100 and 1480 cm^−1^ and bands originating from CH_3_/CD_3_‐coupled modes are colored in orange. For a proper display, the green line spectra in Figure S7 a (in the Supporting Information) represent water complexes of CAME, but they are not considered for CD_3_‐CAME.

It is remarkable that the calculated vibrations of CD_3_‐CAME in Figure S7 b (in the Supporting Information) marked in orange and red have very low intensities, being hardly detected in the experiment. Red and orange monomer peaks are already very weak in the non‐labeled CAME spectrum. The sharp and highly resolved matrix isolation spectra and the consideration and calculation of dimers and water complexes allow a much more comprehensive assignment compared with the analysis of the FTIR spectra of the solid. This was a crucial factor for the misinterpretation by HHM and led—together with the low known solubility and reactivity of KHCO_3_ in alcohols—to the outdated conclusion of different carbonic acid monomers.

Table 4 and Table 5 list all assignable peaks of monomer and dimer structures of CAME and CD_3_‐CAME including H/D shifts but in this context, we want to pick out some characteristic examples to illustrate the challenging spectral appearance in the case of CH_3_/CD_3_ exchange.

Typical pure CH_3_/CD_3_ modes of monomer structure I that shift with a H/D factor of approximately 1.3–1.4 (see Table 4) that are found as very weak signals in the experimental spectra are ν_as_(CH_3_/CD_3_) and ν_s_(CH_3_/CD_3_). The ν(C‐OCH_3_)+δ(CH_3_) mode of monomer I, detected at 1193/1189 cm^−1^ for CAME, splits and bands at 1115 and 905 cm^−1^ can be assigned as pure ν(C‐OCH_3_) and δ(CH_3_) modes for CD_3_‐CAME.

In the direct comparison of the CAME and CD_3_‐CAME matrix isolation spectra in Table 5, no dimer peaks with a typical H/D shift are found. However, the successful isotopic labeling is proven by the overall change of the band positions, especially by change/disappearance of CH_3_/CD_3_‐coupled modes.

Typical examples are δ_ip_(OH)+δ_ip_(CO_3_)+δ(CH_3_) modes of dimers 1, 2, and 3 around 1500 cm^−1^ that are split. The coupled modes are detected at 1486 cm^−1^ in the CAME spectrum and the decoupled modes of δ_ip_(OH)+δ_ip_(CO_3_) and δ(CH_3_) are detected at 1472/1460 and 861 cm^−1^ in the CD_3_‐CAME matrix isolation experiment. ν(CH_3_)+ν(OH) and ν(CH_3_) modes of dimer 1 at 3163/3158 and 3038/3132 cm^−1^ (wavenumber calculated×0.98), which are distinct signals in the CAME spectrum disappear. Upon CH_3_/CD_3_ exchange, the CH_3_‐part is decoupled and one pure theoretically weak ν(OH) remains in the original wavenumber region at 3160 cm^−1^. “New” but weak signals of ν(CH_3_) arise theoretically at 2348, 2326, and 2175 cm^−1^. These peaks are not detected/resolved in the CD_3_‐CAME matrix isolation experiment and have very low intensities in the calculated spectra as well.

#### 2.6.3 Evaluation of the composition in matrix and solid spectra

Whereas monomer bands dominate the matrix isolation spectrum, dimer bands are a better match for the broad bands in the solid‐state spectrum. For example, the characteristic ν(C=O) and δ_ip_(OH) modes of monomer I and II are clearly resolved in the spectrum of the Ar matrix above 1750 and at 1182 cm^−1^ but for the solid‐state spectra the broad characteristic signals around 1720, 1480, and 1310 cm^−1^ results from ν(C=O), δ_ip_(OH)+δ_ip_(CO_3_)+δ(CH_3_), and δ_ip_(OH) dimer modes.

The matrix experiments can be explained based on a ratio of 6:1 between structure I and II, possibly with traces of structure III. The remaining bands can be explained very well based on the presence of cyclic dimers. Specifically, dimers composed of two structure III monomers are identified. This suggests that such building blocks might be present in the CAME polymorph before sublimation. Based on our thermodynamic calculations, CAME dimers are much more likely to occur in the matrix than CA dimers, where no dimers are found.^19^ Of course, the CAME dimers could also exist because of favorable kinetics, that is, a low reaction barrier, for their formation and unfavorable kinetics, that is, a high reaction barrier, for the decomposition into its components.

The 6:1 ratio of monomer structure I and structure II deviates from the ratio in thermodynamic equilibrium on the basis of the PES depicted in Figure 1 b. Thermodynamically, a ratio *K* of 22:1 would be expected, utilizing the relationship Δ*G*=*RT*ln *K* with a sublimation temperature of 210 K and the calculated relative free energy difference of 5.4 kJ mol^−1^ between structure I and II in equilibrium. An equivalent consideration for structure III with a free energy difference of 14.5 kJ mol^−1^ leads to a high ratio, which indicates that it is unrealistic to detect any signals of structure III in thermodynamic equilibrium. This discrepancy to the experimental ratios was already discussed in detail for a similar situation for the matrix experiments of the monoethyl ester of carbonic acid in reference 15. The difference might be caused by the rather short flight time of gas‐phase molecules from the surface of solid CAME at 210 K to being trapped in the Ar matrix at 10 K. In our setup, this flight time is about 0.5 ms. Thus, the monomer ratio might be controlled kinetically, not thermodynamically. An analogous calculation as in ref. 15 (see the Supporting Information) yields a 6:1 ratio of structure I and II in the matrix, which indicates an original ratio of 1:2 sublimating from the crystal.

## 3 Conclusion

The cryo‐preparation and rapid quenching technique complemented with FTIR spectroscopy developed by HHM more than 20 years ago^9^ has proven to be a very suitable tool to prepare and characterize metastable, short‐lived intermediates, in particular H_2_CO_3_ and its derivatives. A large body of significant work, especially on solid H_2_CO_3_, has been published, including studies on the polymorphism of H_2_CO_3_. Two polymorphs of H_2_CO_3_ are described in the literature, namely β‐H_2_CO_3_ 9, 10c, 10d isolated from aqueous solutions and α‐H_2_CO_3_ 10a, 10b, 10d isolated from methanolic solutions. For both polymorphs, the amorphous phase, the crystalline phase, and the transition were described.^11^ Furthermore, the conversion from β‐H_2_CO_3_ to α‐H_2_CO_3_ by dissolving β‐H_2_CO_3_ in MeOH/HCl was reported.10a The reassignment of a hemiester rather than a carbonic acid polymorph in the case of ‘α‐carbonic acid′ was originally proposed in the Ph.D. thesis of our co‐author Jürgen Bernard.^8^ A similar conclusion was made later by Reisenauer et al.^16^ based on a comparison of matrix isolation spectra of a mixture of isobutene/CAME^16^ and matrix isolation spectra of α‐H_2_CO_3_.^12^

In the present study, the rectification of the assignment of FTIR spectra of solid and matrix isolated formerly termed ‘α‐H_2_CO_3_′ is built on four pillars: variation of solvents during different preparation steps, isotopic shifts in the solid‐state spectra, nearly complete clarification of all bands between 4000–600 cm^−1^ of matrix isolation IR spectra supported by MP2/ aug‐cc‐pVTZ calculations, and isotopic labeling and forced conversion of conformers by irradiation experiments of the molecules trapped in the matrix. We used a similar preparation technique as HHM by dissolving KHCO_3_ in absolute methanol followed by cryo‐preparation steps and acidification. The resulting product is the monomethyl ester of carbonic acid (CAME).

The variation of solvents during preparation proves the high reproducibility, purity, and stability of either carbonic acid, CAME, or CAEE. It is decisive which solvent is used in the first preparation step, that is, in water β‐H_2_CO_3_ forms, in MeOH CAME forms, and in EtOH CAEE forms.^15^ Acid‐catalyzed hydrolysis and formation of CA do not take place under these conditions.

By using CD_3_‐labeled MeOH as a solvent, the FTIR spectra of the solid product reveal that the O‐CD_3_ group is transferred from MeOH to the salt and ultimately also to the product, which is CD_3_‐CAME but not α‐H_2_CO_3_. The presence of the methyl group in the product is evidenced by H/D ratios of 1.3–1.4 of the related bands in the spectra. These bands are now reassigned10b as CH/CD modes. We find no evidence of CA in the solid‐state spectra of CAME.

IR spectra obtained after evaporating the solid at 210 K and trapping the vapor in an argon matrix at 10 K can also be reassigned on the basis of CAME in contrast to the former assignment as carbonic acid monomers and dimers.^12^ The assignment relies on isotope substitution experiments, including deuteration of the OH and CH_3_ groups as well as ^12^C and ^13^C isotope exchange. In comparison to our earlier work,^12^ this is of particular relevance to distinguish whether the weak bands with unclear assignment pertain to the terminal methyl group or have any other origin, such as contamination. The experimental study is supported by a comprehensive calculation of high‐level ab initio frequencies for gas‐phase molecules and clusters by using the harmonic approximation. Specifically, three distinct CAME monomer conformers, six distinct CAME dimers, showing the cyclic dimer motif, two 1:1 CAME–H_2_O clusters and two 1:2 CAME–H_2_O clusters were considered. The assignment is based mainly on a comparison of the isotope shifts observed in experiment and calculation. In addition, conformational isomerization is triggered in the matrix through irradiation—so that two monomer conformations can be clearly distinguished by using difference spectroscopy.

On this basis, we find the presence of structure I and structure II monomers in a 6:1 ratio in the matrix. The structure III monomer is found in trace amounts, if at all. In addition, we find a total of about 10 % CAME dimers and CAME–water clusters in the matrix as well as traces of the CAME decomposition product methanol and some CO_2_/H_2_O condensed from the background gas. By contrast to earlier studies, including the work of Reisenauer et al. and our own work,^8^, ^12^ this analysis allows us to explain almost every single band in the whole spectral range between 4000 and 600 cm^−1^, with only a handful exceptions. Although the earlier assignment assuming H_2_CO_3_ shows a good agreement with the observed matrix spectra, the new assignment assuming CAME (HO‐CO‐OCH_3_) represents an excellent agreement, explaining also the weak bands pertaining to the terminal methyl group. Based on this, we define marker bands (Figure S6 in the Supporting Information) distinguishing carbonic acid, CAME, and CAEE. The spectra observed here clearly are not a mixture between CAME and carbonic acid. Rather all bands can be explained without invoking carbonic acid in the new assignment presented here.

Our assignment is much more detailed and comprehensive than the assignment presented by Reisenauer et al. in their Supporting Information Tables S9–S12, which is based merely on two monomer conformers. After two hours of pyrolysis at 920 K, their ratio of structure I/II amounts to 2:1 as judged from the intensity ratio of the 1776/1826 bands in their Figure 3 c. This corresponds to the situation expected in thermodynamic equilibrium: for Δ*G*=5.4 kJ mol^−1^ and *T*=920 K, a ratio of 2:1 for structures I and II is expected, *K*(I/II)=exp(Δ*G*/*RT*)=2.0. However, Reisenauer et al. have overlooked structure III in their spectra. Based on Δ*G*=14.5 kJ mol^−1^ and *T*=920 K, the ratio between structure I and structure III should be about 6.4 in thermodynamic equilibrium, *K*(I/III)=exp(Δ*G*/*RT*)=6.4. In fact, in Figure 3 c of Reisenauer et al. an unmarked band near 1800 cm^−1^ appears that has about 1/6 of the intensity of the band at 1776 cm^−1^. This band is exactly at the position predicted from our calculations for structure III. Based on the shifts between structures I and III and the intensities calculated here, we would expect the bands for structure III at the following positions. In brackets, we provide the expected intensity compared with the structure I peak for an assumed ratio of I/III=6:1, 3592 (1/6 of 3611), 1803 (1/6 of 1776), 1459 (1/24 of 1452), 1353 (1/6 of 1383), 1197 (1/36 of 1182), 1130 (1/12 of 1182), 880 (1/36 of 899), and 780 (1/6 of 794). Unfortunately, these regions are not shown in the work by Reisenauer et al., except for the band at 1803 cm^−1^ that is observed. This implies a mixture of three CAME monomers I/II/III=6:3:1 after high‐temperature pyrolysis at 920 K, compared with a ratio of 6:1:0 after low‐temperature sublimation at 210 K here. Whether or not dimers are present in the spectrum by Reisenauer et al. is hard to judge because important bands are obscured by the pyrolysis products isobutene and *t*‐butanol and unreacted *tert*‐butyl methyl carbonate in their spectra. In our spectra, we identify about 10 % of all molecules to be dimers. Presumably, this reflects the fact that the solid releases CAME dimers upon sublimation at 210 K, which do not have enough time to decompose in the 0.5 ms flight time before being trapped in the matrix. Compared with H_2_CO_3_ dimers, CAME dimers have higher dimerization energies, and the decomposition of the monomer into CO_2_ and CH_3_OH is less favored. Besides these thermodynamic arguments, CAME dimers may also exist owing to kinetic stabilization. Traces of methanol detected in the matrix spectra may suggest that a small part of CAME is in fact decomposed.

Taking the solid‐state spectra and the matrix spectra together removes the basis for the existence of α‐H_2_CO_3_ and carbonic acid polymorphism. The previously reported transition from amorphous to crystalline α‐H_2_CO_3_ is still valid, but has to be reinterpreted as the transition from amorphous to crystalline CAME.^11^ The previously reported polymorphic transition from β‐ to α‐H_2_CO_3_ 10a has to be reinterpreted as a methylation of β‐H_2_CO_3_ in acidic methanolic solution. The matrix spectra of H_2_CO_3_ isolated from ′α‐carbonic acid′^12^, ^19^ are reinterpreted as well. This conclusion could not have been made without the isotopic labeling studies and the comparison with high‐level calculations for a comprehensive set of molecules. Only this combination of experimental and theoretical studies has allowed us to confidently reassign the weak bands originating from the CH_3_ group. Rather than the previous good agreement reached by Hage, Hallbrucker, and Mayer for the solid^9^–, 10d and by Bernard et al.^12^ for the matrix spectra, an excellent agreement has been reached in this work for both. The CAME polymorph seems to preferentially contain dimeric units composed of structure III. However, the crystal structure remains unknown and needs to be determined in future work. After this reinterpretation, the only polymorph of carbonic acid that remains is the β‐polymorph crystallized from aqueous solution10c or produced by irradiation of CO_2_/H_2_O mixtures^13^ or proton implantation of CO_2_ ices.13b, 27 A γ‐polymorph might have been produced in the work by Oba et al. from the reaction of CO with OH radicals.^28^ However, the existence of γ‐H_2_CO_3_ is speculative currently and needs to be established in future work.

## Experimental Section

### Preparation experiment in the solid state

The preparation of the starting material was done from methanolic solution of potassium bicarbonate (KHCO_3_, Sigma–Aldrich, >99.5 %). Alkali bicarbonates and carbonates are barely soluble in methanol, whereas they can easily be dissolved in water. KHCO_3_ was stirred in CH_3_OH (Sigma–Aldrich; methanol CHROMASOLV®, for HPLC, ≥99.9 %) or CD_3_OH (Sigma–Aldrich; methanol‐D3, 99.8 atom % D). KHCO_3_ has a p*K*_a_ of 10.25 and CH_3_OH has a p*K*_a_ of 15.5.^7^, ^29^ Complementary experiments were done by using doubly distilled, deionized H_2_O or absolute ethanol as solvents. The solutions were nebulized in N_2_ carrier gas by means of an air brush pistol (Harder & Steenbeck; model grafo or infinity) and introduced into a vacuum chamber (≈10^−7^ mbar) through an aperture (500 μm). Upon impact of the aerosol on a cryoplate at liquid nitrogen temperature (*T*=78 K), a layer of glassy solution forms. The solution droplets (>10 μm in diameter) are immobilized almost instantaneously at cooling rates up to 10^5^ K s^−1^.^30^ IR transparent windows (cesium iodide, CsI, or silicon, Si, windows) serve as the cryoplate.^9^, 10d After deposition of the bicarbonate solution, the cryoplate was heated in vacuo to 290 K, which results in evaporation of the solvent and a solid precipitate remaining on the cryoplate. This step was not part of the protocol employed by HHM.^9^, ^10^ The solid precipitate was later protonated by depositing a layer of glassy 1.5 m HCl (diluted from Supelco methanolic HCl 3 N or hydrochloric acid HCl 37 %) solution, either in water, methanol, or ethanol at *T*≈80 K and subsequent heating. Heating triggers diffusive mixing of the acid with the base, acid–base reaction, and finally evaporation of solvent.

The reactions were monitored in situ by Fourier transform infrared (FTIR) spectroscopy by using a Varian Excalibur 3100, in which the beam of light passes through optical windows (KBr) into the vacuum chamber, through the thin film sample and out of the vacuum chamber to the detector. FTIR spectra were recorded with a resolution of 4 cm^−1^ and by accumulating 100 scans. The chamber was pumped to a base pressure of 10^−7^ mbar by using an oil‐free scroll pump (Varian Triscroll) and a turbomolecular pump (Leybold Turbovac 361). To keep the base pressure after the injection of the nebulized solutions in nitrogen as carrier gas low, a cryopump (Leybold RW 6000 compressor unit and RGD 1245 cold head) was located inside the vacuum chamber and kept at 11 K. At this temperature, the carrier gas condenses as a solid on the cryopump.

### Experiment in the gas phase

After preparation of the pure solid in the laboratory in Innsbruck, the cryoplate containing the CAME film was removed from the vacuum chamber, immersed in liquid nitrogen, and transported to Vienna for matrix isolation experiments. In Vienna, an ultrahigh‐vacuum chamber was used, which was previously employed for successfully isolating reactive species such as halogen oxides^31^ or carbonic acid.^32^ FTIR spectra were recorded by using a Bruker Vertex 80v, which offers an evacuated optical path (2 mbar) and a resolution of 0.2 cm^−1^ at which 1024 scans were accumulated. The details of the matrix isolation procedure can be found in reference 12.

UV irradiation experiments were performed by using an Hg(Xe) arc light source operated at 300 W (solar simulator AM1.5G). While irradiating for 10 min, the matrix temperature was kept below 10 K. Subsequent to UV irradiation, FTIR spectra were collected and analyzed as difference spectra compared with measurements prior to irradiation. The sharp bands observed in matrix isolation spectra with a very high resolution are perfect for monitoring isotope shifts and a direct comparison with calculated gas‐phase spectra.

Just like for mass‐spectrometric techniques, evaporation of the solid is required for matrix isolation spectroscopy. However, ionization and ionization‐induced fragmentation are not an issue in the matrix isolation technique as the neutral molecules are landed in the matrix, by contrast to mass‐spectrometric techniques. In other words, as carbonic acid and carbonic acid esters very readily fragment upon ionization, both of them show fragments at the same *m*/*z* ratio, and this technique is not suitable for discriminating between CAME and carbonic acid. Also, the presence or absence of the methyl group in the mass spectrum cannot be reliably used to discriminate between the two as traces of methanol may be present in carbonic acid, for example, as inclusion. By contrast, the vibrational bands are shifted between the two molecules, and so matrix isolation spectroscopy is the better analytic technique to assess the purity of the sample. X‐ray diffraction would in principle be suitable as well. However, the thin film nature of the samples and their in situ formation in a vacuum chamber do not allow for a ready investigation by diffraction.

### Quantum chemical setup

The low‐energy conformations of carbonic acid methyl ester (CAME) were determined by second‐order Møller–Plesset perturbation theory (MP2)^33^ by using augmented correlation consistent basis sets by Dunning and co‐workers of triple‐zeta quality (aug‐cc‐pVTZ).^34^ To estimate the kinetics of conformational interconversion, a relaxed potential energy surface (PES) scan was performed for the two dihedral angels that need to be rotated to interconvert the four conformers. The O=C‐O‐H and O=C‐O‐CH_3_ dihedrals were scanned at 15° intervals from 0° to 345° and 0° to 180°, respectively. A total of 312 points were calculated at MP2/aug‐cc‐pVTZ level of theory and the resulting PES is shown in Figure 1 b. The PES energies do not include zero‐point corrections, which were shown to be negligible previously for CAEE^15^ and are also negligible here. All of these calculations were done in *C*_1_ symmetry and performed by using Gaussian 09 Rev. C01.^35^

The three low‐energy monomer conformers were combined to construct six dimer structures, which were structure optimized by using MP2/aug‐cc‐pVTZ. For these calculations, molecular symmetries had to be exploited. For consistency, the three low‐energy monomers of CAME were re‐optimized with MP2/aug‐cc‐pVTZ when applying their molecular symmetry group. These calculations were performed with Turbomole 7.1.1.^36^ Energy differences between the fully symmetric and *C*_1_ symmetric molecules are negligible. Dimerization energies and interaction energies were calculated on the MP2/aug‐cc‐pVTZ optimized structures as single points by using the explicitly correlated coupled cluster variant CCSD(T)‐F12^37^ with density fitting—as implemented in Molpro 2015.1^38^—in combination with a triple‐zeta basis‐set (cc‐pVTZ‐F12).^39^ Thermal as well as zero‐point energy corrections to obtain dimerization and interaction free energies at temperatures between 180 and 220 K were obtained by MP2/aug‐cc‐pVTZ. A number of CAME–water complexes with one or more water molecules at different positions were subjected to quantum chemical structure optimization. However, only those up to 20 kJ mol^−1^ were used for the further studies.

Normal modes and infrared intensities for annotating the measured spectra were obtained for three distinct conformations of CAME, for three CAME–water clusters, and for three low‐energy CAME dimers (dimers 1–3) by using the harmonic approximation at the MP2/aug‐cc‐pVTZ level of theory. For each conformation, only real frequencies were obtained, confirming all investigated structures to be minimum energy conformations. Thermal and zero‐point energy corrections were calculated at 210 K (the experimental temperature) and *p*=2×10^−5^ mbar, with frequencies scaled by a factor of 0.9792 as suggested by Kesharwani et al.^40^

For each of the three CAME low‐energy conformers, isotopic shifts for the three isotopically labeled variants ^13^C‐CAME, CD_3_‐CAME, and OD‐CAME were extracted by transformation of the reduced masses in the mass‐weighted Hessian matrix, which is computed at the MP2/aug‐cc‐pVTZ level of theory by numerical second derivatives. For the three low‐energy conformations of the CAME dimers, shifts for all singly and double isotopically labeled species were obtained, resulting in the following variants: dimer 1 and dimer 3: ^13^C‐^13^C, ^13^C‐^12^C, CD_3_‐CD_3_, CD_3_‐CH_3_, OD‐OD, OD‐OH (six isotopically labeled species for each dimer); dimer 2: ^13^C‐^13^C, ^13^C‐^12^C, ^12^C‐^13^C, CD_3_‐CD_3_, CD_3_‐CH_3_, CH_3_‐CD_3_, OD‐OD, OD‐OH, OH‐OD (nine isotopically labeled species). Intensities of the isotopically labeled species were scaled according to their experimentally determined abundance in the sample. All these calculations were performed with Molpro 2015.1.^38^

This extensive analysis allowed us to identify the various convoluted signals observed in the experimentally obtained spectra. Vibrational spectra were scaled by the factor of 0.98 to match the experimentally observed C‐O stretch mode. According to the “Computational Chemistry Comparison and Benchmark DataBase”, the best scaling factor to be used for vibrations calculated by using MP2/aug‐cc‐pVTZ is 0.953±0.033 as determined from a comparison of 358 vibrations in 117 molecules.^32^ Structures were visualized with VMD.^41^

## Conflict of interest

The authors declare no conflict of interest.

## Supporting information

As a service to our authors and readers, this journal provides supporting information supplied by the authors. Such materials are peer reviewed and may be re‐organized for online delivery, but are not copy‐edited or typeset. Technical support issues arising from supporting information (other than missing files) should be addressed to the authors.

Supplementary
