# Supplementary material for: Alpha‐Carbonic Acid Revisited: Carbonic Acid Monomethyl Ester as a Solid and its Conformational Isomerism in the Gas Phase
Source: Chemistry. 2019 Nov 26;26(1):285–305. doi: 10.1002/chem.201904142 (PMC6972543; doi:10.1002/chem.201904142)
Supplement: Supplementary file 1 — Supplementary [file CHEM-26-285-s001.pdf]

CHEMISTRY
A European Journal

Supporting Information

Alpha-Carbonic Acid Revisited: Carbonic Acid Monomethyl Ester as a Solid and its Conformational Isomerism in the Gas Phase

Eva-Maria Köck,[a, b] Jürgen Bernard,[a, b] Maren Podewitz,[c] Dennis F. Dinu,[c] Roland G. Huber,[c] Klaus R. Liedl,[c] Hinrich Grothe,*[d] Erminald Bertel,[a] Robert Schlögl,[b] and Thomas Loerting*[a]

chem_201904142_sm_miscellaneous_information.pdf

Preparation and FT-IR characterization of solid CAME

Reaction Pathway

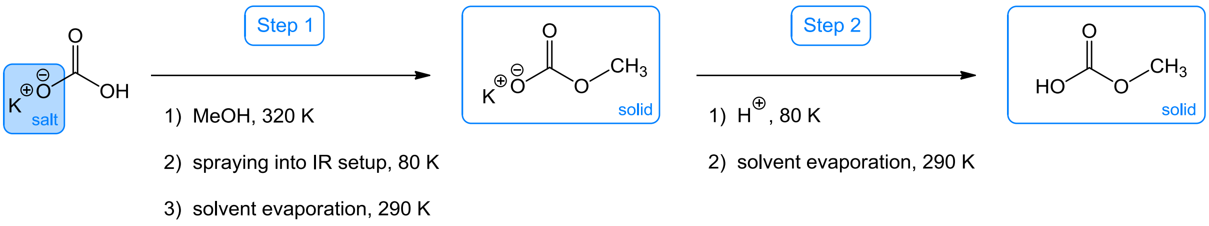

Figure S1: Preparation of monomethyl carbonate (step 1) and carbonic acid monomethyl ester (step 2) from solid $KHCO_3$ in water-free methanol.

The preparation procedure of CAME illustrated in Figure S1 can be divided into two steps: (1) monomethyl carbonate formation and (2) formation of CAME. Step (1) starts by immersing and stirring solid $KHCO_3$ salt in water-free liquid methanol at 320 K. $KHCO_3$ is barely soluble in methanol. We assume that addition of methanol followed by the elimination of water takes place in solution at very low concentrations in the solution itself or near the solid-liquid interface. The elimination of water is promoted by the hygroscopic effect of water-free methanol. Furthermore, the resulting potassium monomethyl carbonate $K[O_2COCH_3]$ is slightly more soluble than $KHCO_3$[1]. It is unclear whether a mixture of both the methylated and unmethylated salt is present in equilibrium at 320 K or whether all of the dissolved bicarbonate has already transformed to monomethyl carbonate. Next, a glassy layer is formed by spraying the $K[O_2COCH_3]$ solution onto the cryoplate kept at 80 K, which is subsequently heated to 290 K. By pumping off the solvent (CH_3OH) during heating to 290 K a solid residue of $K[O_2COCH_3]$ remains. Evaporation of the solvent methanol and the better solubility of the methylated salt would force the reaction to completion in case an equilibrium mixture was present in the glassy solution. The corresponding FT-IR spectrum in Figure S2a provides clear evidence of the monomethyl carbonate, without any signs of $KHCO_3$. None of our spectra show evidence for residual $KHCO_3$.

In step (2) protonation/acidification, is triggered inside the vacuum chamber at cryoconditions. This is achieved by depositing a layer of glassy acid solution (such as aqueous HCl) on top of the solid. Upon heating the acid becomes diffusive above the glass transition temperature of the solution (~ 140 K), then acid-base reaction takes place, yielding CAME in solution (FT-IR spectrum in Figure S2b). The solvent can again be pumped off since its vapor pressure is higher than CAME’s vapor pressure, leaving solid CAME behind. Typically, the solvent evaporates between 190 and 220 K in our setup, depending on the type

of solvent and the background pressure. The solid is initially amorphous and may crystallize upon further heating.

Figure S2a shows the FT-IR spectrum of the solid residue, which was obtained after CH_3OH removal. Figure S2b shows the FT-IR spectrum of the solid after the subsequent treatment with acid. A comparison with literature reveals a close resemblance between the spectrum seen in Figure S2a and the spectrum of potassium monomethyl carbonate ($K[O_2COCH_3]$) reported by Behrendt *et al.* in 1973.[2] The left column in Table S1 demonstrates the good agreement by listing the band positions reported by Behrendt *et al.* and the band positions extracted from Figure S2a. We agree with all assignments given by Behrendt *et al.* with the exception of the band at 1186 cm^{-1}. Based on our isotope substitution experiments we reassign this band to be a $\delta(CH_3)$ mode rather than $\nu(CO)$. Judging from the relatively broad and symmetric bands in Figure S2a as compared to the more featured and split bands by Behrendt *et al.* we assume an amorphous or partly crystalline solid in our case and a crystalline solid in their case. They had prepared the potassium salts of carbonic acid methyl- and ethyl-hemiesters by reaction of CO_2 with alcoholates and characterized the salts using FT-IR, UV and ^{1}H-NMR spectroscopy [1-3]. In 1994 Adam and Cirpus obtained $K[O_2COCH_3]$ from reaction of dimethyl carbonate with hydroxide in methanol. $K[O_2COCH_3]$ forms long, flattened and colorless needles, which are sensitive to moisture[4]. The crystal structure was determined as triclinic $P\overline{1}$, $Z = 2$, a = 3.809(2) Å, b = 5.589(3) Å, c = 9.853(3) Å, α = 100.71(2)°, β = 90.06(3)°, γ = 92.48(3)°.[4]

After acidification of $K[O_2COCH_3]$ we obtain CAME (HO_2COCH_3). Table S1 lists the assigned frequencies of the bands in Figure S2b. The literature spectrum which resembles the one in Figure S2b the best is a spectrum of "α-H_2CO_3" (prepared with the technique of HHM) where the solid residue was forced to crystallize by an annealing procedure[5]. Even though the preparation technique by HHM was different (see introduction), it seems they have prepared the same solid as obtained also by us. We, therefore, reinterpret their spectra on the basis of our findings.

Compared to the interpretation by HHM[6] on the basis of α-H_2CO_3 the values in red in Table S1 are the newly interpreted frequencies: The bands at 2990, 2918 and 2880 cm^{-1} can be assigned to $\nu(CH_3)$ modes, the bands at 1464/1447/1423 cm^{-1} as well as at 1325 and 1312 cm^{-1} correspond to modes coupled with $\delta(CH_3)$ vibrations– indicating the presence of the methyl ester group. The band at 1200 cm^{-1} is a pure $\delta(CH_3)$ vibration and the one at 1086 cm^{-1}

corresponds to a ν(O-CH_3) mode. While the spectrum of the monomethyl ester in Figure S2b is very similar to the one of α-H_2CO_3 it clearly differs from the spectrum of β-H_2CO_3.

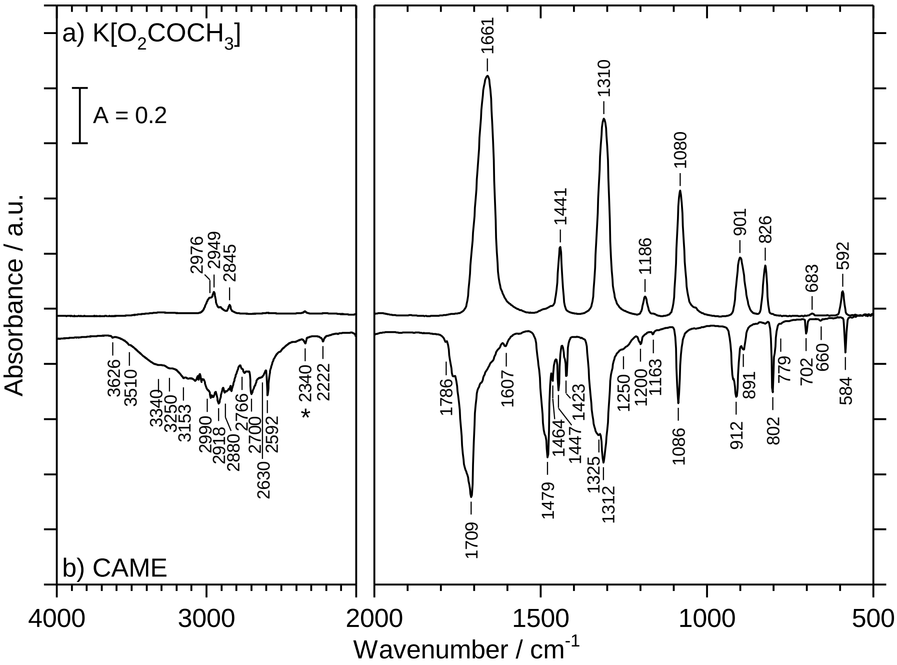

Figure S2: a) FT-IR spectrum of solid K[O_2COCH_3]. The spectrum was recorded *in vacuo* at 290 K. b) FT-IR spectrum of solid CAME (HO_2COCH_3) after protonation of solid K[O_2COCH_3] with HCl in H_2O, recorded at 80 K. The band marked with an asterisk belongs to CO_2. The negative absorbance is a result of a mathematical operation (multiplication with -1), facilitating comparison.

Table S1: Assignment of the IR frequencies of solid $K[O_2COCH_3]$ and solid HO_2COCH_3. Indicated in bold red are newly assigned bands (all values in cm^{-1}).

**$K[O_2COCH_3]$**			**HO_2COCH_3**		**α-H_2CO_3**	
**expt. Figure S2a**	**Behrendt *et al.* [2]**	**Assign. [2]**	**expt. Figure S2b**	**Assign.**	**HHM [6]**	**Assign.[6]**
			3626<br>3510<br>~3340<br>~3250<br>3153	$\nu(OH)$		
2976 (sh)<br>2949 (w)	3003 (w)<br>2967 (w)	$\nu_{as}(CH)$	**2990**<br>**2918**<br>**2880**	**$\nu(CH_3)$**	3024	combination bands
2845 (vw)	2857 (w)	$\nu_s(CH)$	**2766, 2747**	**$\nu(CH_3)$, $\nu(OH)$**		
			2700	$\nu(OH)$	2694	$\nu(OH)$
			**~2675**<br>**~2630**	**$\nu(CH_3)$, $\nu(OH)$**		
			2592	$\nu(OH)$	2585	$\nu(OH)$
			**1786**	**$\nu(C{=}O)$**		
1661 (s)	1689 (s)	$\nu_{as}(C{=}O)$	1709	$\nu(C{=}O)$	1715	$\nu(C{=}O)$
			1607	$\delta(OH)$		
			**1479**	**$\nu(C\text{-}OH)$**	1477	$\nu_{as}[C(OH)_2]$
1441 (m)	1475 (m)<br>1453 (s)<br>1435 (s)	$\delta_{as}(CH_3)$	**1464**<br>**1447**<br>**1423**	**$\delta_{ip}(OH)$, $\delta_{ip}(CO_3)$, $\delta(CH_3)$**	1457<br>1420	disordered H_2CO_3<br>$\delta_{ip}(COH)$
			**1325**<br>**1312**	**$\delta_{ip}(OH)$, $\delta_{ip}(CO_3)$, $\delta(CH_3)$**	1323<br>1304	$\nu_{as}[C(OH)_2]$
1310 (s)	1305 (s)	$\nu_s(CO_2)$				
	1282 (sh)	$\delta_s(CH_3)$	**~1250**	**$\nu(C\text{-}OH)$**	~ 1250	disordered H_2CO_3
1186 (w)	1185 (m)<br>1163 (w)	**$\delta(CH_3)$/**<br>$\nu(CO)$	**1200**	**$\delta(CH_3)$**	1199	
			**1163**	**$\delta(CH_3)$**		
1080 (s)	1079 (s)	$\nu(CO)$	**1086**	**$\nu(O\text{-}CH_3)$**	1084	$\nu_s[C(OH)_2]$
901 (m)	893 (s)	$\nu(CH_3O)$	**912**<br>**891**	**$\delta_{ip}(C\text{-}O\text{-}CH_3)$**<br>**$\delta_{oop}(OH)$**	920	$\delta_{oop}(COH)$
826 (m)	826 (s)	$\delta_{oop}(CO_3)$	802<br>779	$\delta_{oop}(CO_3)$	801	$\delta_{oop}(CO_3)$
683 (vw)	672 (w)	$\delta_{as}(CO_2)$	**702**<br>**660**	**$\delta_{ip}(CO_3)$**	705<br>699	
592 (w)	589 (m)	$\delta_s(CO_2)$	584	$\delta_{ip}(CO_3)$	583	$\delta_{ip}(CO_3)$

vw, very weak; w, weak; m, medium, s, strong; sh, shoulder
ν_s and ν_{as}, symmetric and asymmetric stretching mode; δ_{ip} and δ_{oop}, in-plane and out-of-plane bending mode; δ_s and δ_{as}, symmetric and asymmetric bending mode

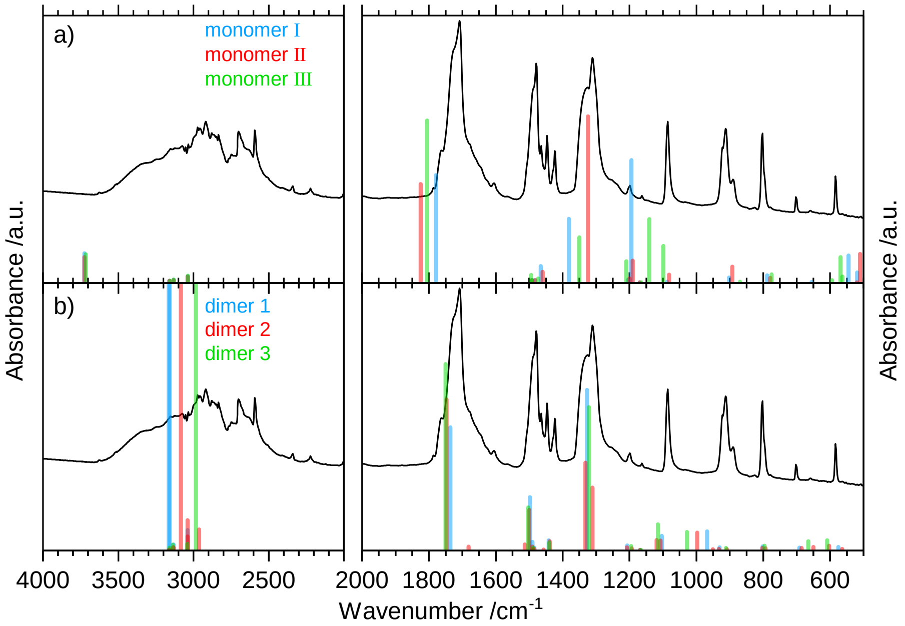

Figure S3: FT-IR spectrum of solid HO_2COCH_3 after protonation of solid $K[O_2COCH_3]$ with HCl in H_2O, recorded at 80 K compared to calculated line spectra of a) monomer I = blue, monomer II = red and monomer III = green and b) dimer 1 = blue, dimer 2 = red and dimer 3 = green.

In order to interpret the matrix isolation spectra after subsequent evaporation of the solid different energetically reasonable conformers of monomers and their dimers are calculated *via* MP2/aug-cc-pVTZ. The match of calculated line spectra of individual molecules *in vacuo* and matrix isolation spectra can be excellent, whereas a comparison with solid state spectra has to be taken with caution. In order to take into account for possible isomerism and dimerization in solid CAME we take into account several CAME monomer as well as CAME dimer conformers. In Figure S3a calculated line spectra of monomers I, II and III are plotted, and in Figure S3b line spectra for three dimers 1 (monomer I + monomer I), 2 (monomer I + monomer III) and 3 (monomer III + monomer III) are displayed. These where chosen based on their thermodynamic stabilities in the gas phase. All dimers feature strong hydrogen bonds (see part 2 for structures). It is obvious that no suitable match for monomers is gained in Figure S3a for the solid CAME spectrum. Monomer I would match some bands to a certain extent, *e.g.* the ν(OH) signal at 3626 cm^{-1}, a very small shoulder at 1786 cm^{-1} (ν(C=O)) of the broad signal at 1709 cm^{-1} together with a signal at 1200 cm^{-1} (δ_{ip}(OH)), but several strong signals *e.g.* at 1479 and 1312 cm^{-1} do not match the calculated data of monomers. The match of calculated and experimental spectra in Figure S3b is more plausible. From the intensities of the ν(OH) (+ ν(CH_3)) dimer bands in the experimental spectrum at 2592, 2700 and 2747/2766 cm^{-1} – corresponding to calculated wavenumbers at 2982, 3083 and 3158/3163 cm^{-1} – one may infer that structure III and structure II dimers are more likely than structure I in the solid.

Variation of the solvent in step (1)

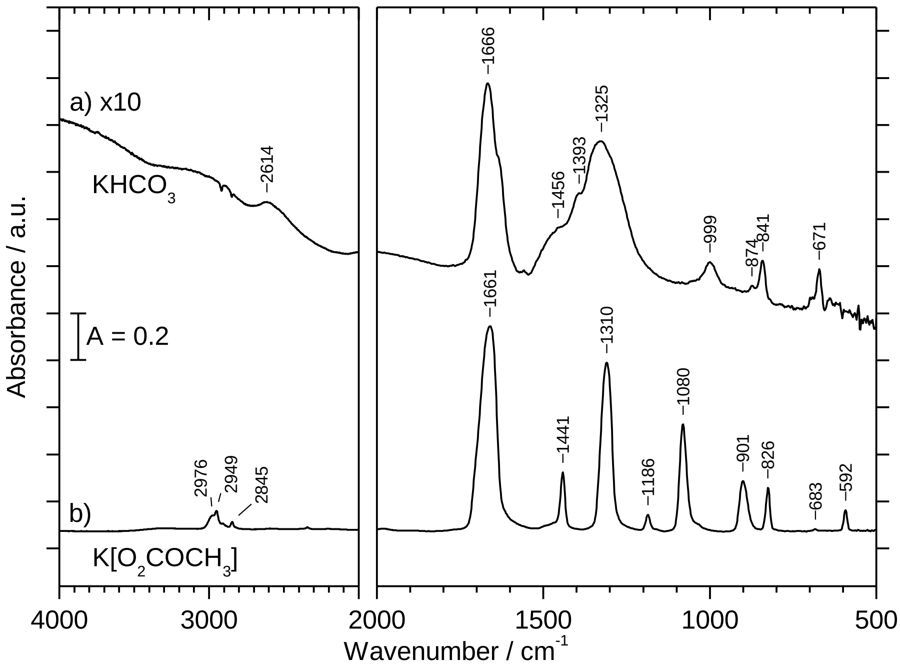

Figure S4: a) FT-IR spectrum of solid $KHCO_3$ isolated from an aqueous solution *in vacuo* at 290 K. b) FT-IR spectrum of solid $K[O_2COCH_3]$, recorded *in vacuo* at 290 K.

Table S2: Assignment of the IR frequencies of solid $KHCO_3$ (all values in cm^{-1})

**$KHCO_3$** Nakamoto *et al.* [7]	**expt., Figure S4a**	**Assign.**
2620 (w)	2608 (br)	ν(O-H)
1618 (s)	1666 (s)<br>1640 (sh)	ν(C=O)
1405 (s)	1456 (sh)<br>1393 (sh)	δ_{ip}(OHO)
1367 (s)	1325 (s, br)	ν(C-O) + ν(C=O) + δ_{ip}(OHO)
1001 (m)	999 (m)	ν(C-O) + ν(C=O)
988 (m)		δ_{oop}(OHO)
830 (m)	841 (m)	$\delta_{oop}(CO_3)$
698 (m)	698 (vw)	δ_{ip}(C=O) + ν(O-H)
655 (m)	671 (m)	$\delta_{ip}(CO_3)$

vw, very weak; w, weak; m, medium, s, strong; br, broad; sh, shoulder
ν, stretching; δ_{ip}, in-plane; δ_{oop}, out of plane bending

A direct comparison of $K[O_2COCH_3]$ and $KHCO_3$ is provided in Figure S4, which shows the FT-IR spectra of a) solid $KHCO_3$ (water as solvent for step (1)) and b) solid $K[O_2COCH_3]$ (methanol as solvent for step (1)). The corresponding bands of $KHCO_3$ and their assignment

are compared to literature values in Table S2. A comparison with the spectrum of Nakamoto *et al.* (Table S2) for crystalline $KHCO_3$.[7] shows clear differences with the hemiester salt.

The spectrum of the residue in Figure S4a is in reasonable agreement with the spectrum of crystalline $KHCO_3$ reported by Nakamoto *et al.*.[7] $KHCO_3$ grows as a monoclinic crystal, space group $P2_1/c$ (a = 15.1725 Å, b = 5.6283 Å, c = 3.7110 Å, β = 104.631°)[8]. In Figure S4a the most intensive band is located at 1666 cm^{-1} and has a shoulder at about 1640 cm^{-1}. The second most intensive band is broad with a maximum at 1325 cm^{-1} and two shoulders at 1456 and 1393 cm^{-1}. All these bands are at similar positions, but shifted compared to Nakamoto *et al.*[7], whereas the bands at 999, 841 and 671 cm^{-1} are in reasonable agreement (see Table S2). Most likely, the band shift and broadness of bands in our spectrum indicates the presence of amorphous rather than crystalline $KHCO_3$. However, the difference between the spectra of $K[O_2COCH_3]$ in Figure S4b and $KHCO_3$ in Figure S4a is obvious. In both cases the preparation routine was exactly the same – the only difference is the solvent in preparation step (1), which was removed before recording the spectra in Figure S4. Therefore, we conclude that amorphous $KHCO_3$ is the product after dissolution in water and subsequent solvent evaporation, whereas amorphous $K[O_2COCH_3]$ is obtained after dissolution in methanol and subsequent solvent evaporation. There are no signs for the presence of $KHCO_3$ in the latter case. Since we are able to resolve bands that are of about 1% of the intensity of a band of medium intensity our detection limit for traces of $KHCO_3$ can be estimated as < 1%. The absence of *e.g.* the bands at 1393, 999, 841 or 671 cm^{-1} indicates that $KHCO_3$ is not detected in Figure S4b, *i.e.*, methylation is complete. Similarly, we have shown in our earlier work that $K[O_2COC_2H_5]$ forms (without any traces of $KHCO_3$) after dissolving $KHCO_3$ in ethanol followed by evaporation of the solvent[9].

Isotope labelling, solid CAME

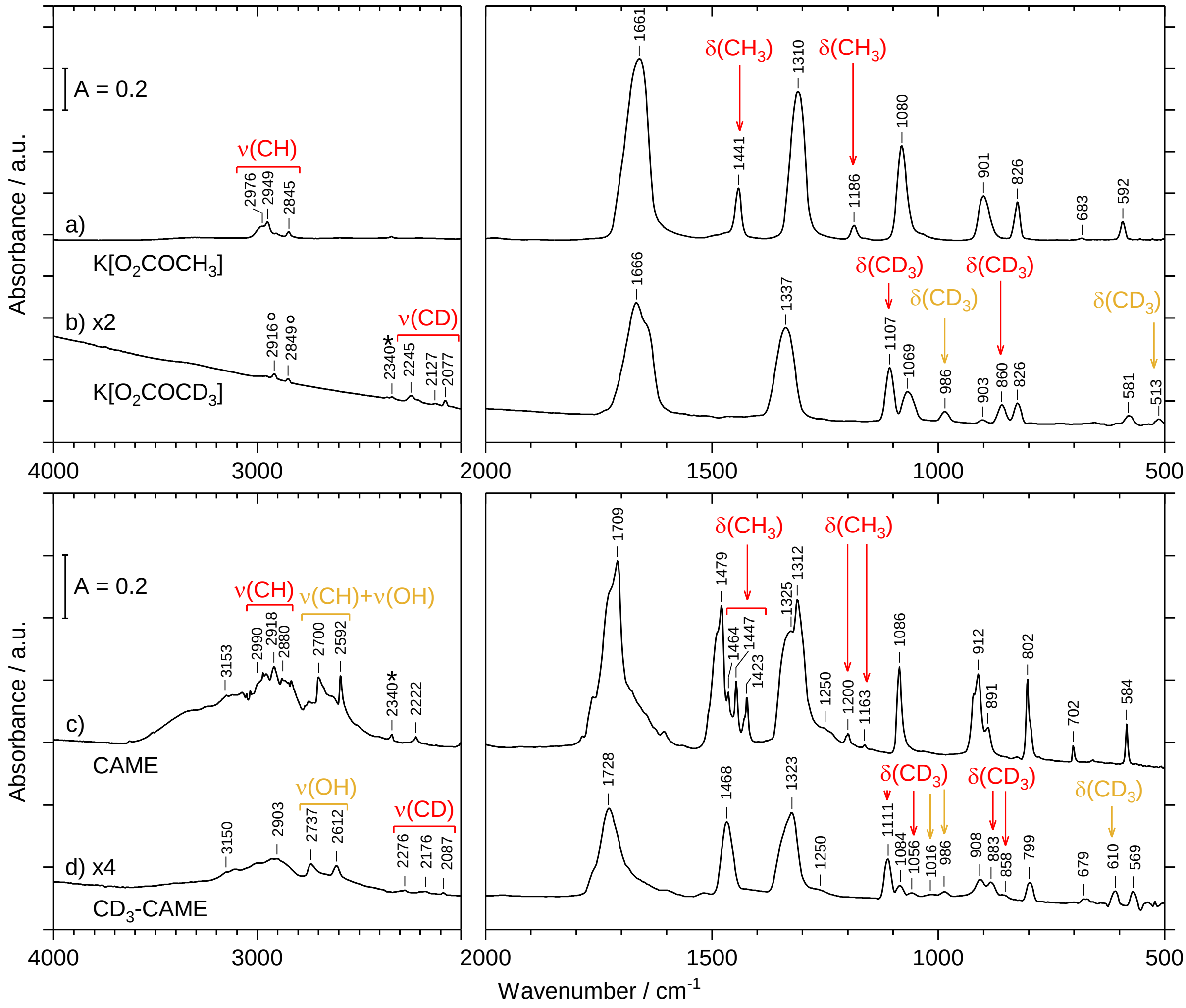

Figure S5: a) FT-IR spectrum of solid $K[O_2COCH_3]$ recorded at 290 K. b) FT-IR spectrum of solid $K[O_2COCD_3]$ recorded at 290 K. c) FT-IR spectrum of solid HO_2COCH_3 recorded at 80 K. d) FT-IR spectrum of solid HO_2COCD_3 recorded at 80 K. The band marked with an asterisk belongs to CO_2, the one with a circle to impurities on the cryoplate. Pure CH_3 vibrational modes are labeled in red and modes that are decoupled upon isotopic labelling are labeled in orange.

Figures S5a and S5b show the FT-IR spectra of the solid K-salt residues after the first evaporation of the solvent at 180 K *in vacuo* (step 1) from CH_3OH and CD_3OH, respectively. Upon comparison it is most notable that especially the bands assigned to the CH_3-group clearly shift.

The modes that shift (marked in red in Figure S5a and S5b and in Table 2) are $\nu_{as}(CH)/\nu_{as}(CD)$ from 2976 and 2949 to 2245 and 2127 cm^{-1}', $\nu_s(CH)/\nu_s(CD)$ from 2845 to 2077 cm^{-1}, $\delta(CH_3)/\delta(CD_3)$ from 1441 to 1107 cm^{-1} and from 1186 to 860 cm^{-1}. The shift factors of all these bands are 1.3 – 1.4, close to $\sqrt{2}$. The coupled mode ν(C-OC)+$\delta(CH_3)$ of $K[O_2COCH_3]$ at 1310 cm^{-1} splits into signals at 1337 and 986 cm^{-1} which is labeled in orange in Figure S5b and Table 2. In the spectrum of $K[O_2COCD_3]$ a band at 513 cm^{-1} is present which we assume to be the $\delta(CD_3)$ vibration, previously assigned as $\delta_{as}(CO_2)$ in Behrendt *et*

al.[2]. The observation of bands at the expected positions after isotope substitution corroborates our finding that in methanolic solution $KHCO_3$ reacts to $K[O_2COCH_3]$ as proposed in Figure S1.

Water complexes, matrix isolation

Table S3: Assignment of IR frequencies of H_2O complexes 1, 2 and 3 of CAME (all values in cm^{-1})

**CAME**		**Assign.**		
Ar	Calc.	Complex	Molecule	Mode
2956*#	3094	2	CAME	ν(OH)
1797*#	1819	3	CAME	ν(C=O)
1722*#	1741	1	CAME	ν(C=O)
1715#	1727	2	CAME	ν(C=O)
1481/1478*#	1500	2	CAME	$\delta_{ip}(CO_3)$, $\delta(CH_3)$, $\delta_{ip}(OH)$
1465/1463*	1482	1	CAME	$\delta_{ip}(CO_3)$, $\delta(CH_3)$
1445*	1461	3	CAME	$\delta(CH_3)$
1389*	1408	3	CAME	$\delta_{ip}(OH)$
1276*	1311	2	CAME	$\delta_{ip}(CO_3)$, $\delta_{ip}(OH)$
1272/1269*	1302	1	CAME	$\delta_{ip}(CO_3)$, $\delta_{ip}(OH)$
1228	1244	3	CAME	$\delta_{ip}(CO_3)$
1216*#	1205	1, 2	CAME	$\delta(CH_3)$
1202*#	1196	3	CAME	$\delta(CH_3)$
1092*#	1007	2	CAME	$\nu(O\text{-}CH_3)$
1079*#	1100	1	CAME	$\nu(O\text{-}CH_3)$
	1082	3	CAME	$\nu(O\text{-}CH_3)$
948	973	2	CAME	$\delta_{oop}(OH)$
933	889	3	CAME	$\nu(C\text{-}OCH_3)$
924*	879	2	H_2O	$\delta_{oop}(O\text{-}H\ \text{---}\ OH_2)$
920, 917*	864	1	CAME	$\delta_{oop}(OH)$
808	823	3	CAME	$\delta_{oop}(OH)$
701	685	2	H_2O	$\delta_{oop}(O\text{-}H\ \text{---}\ O{=}C)$
645	633	1	H_2O	$\delta_{oop}(H\text{-}OH)$
$\bar{x}_{th\text{-}exp<2000\ cm\text{-}1}$	24.9			

Factor calc. = 0.98
ν = stretching mode, δ = bending mode, ip = in plane, oop = out of plane
* *CAME impurity in OD-CAME,* # *CAME impurity in* ^{13}*C-CAME*
$\bar{x}_{th\text{-}exp<2000\ cm\text{-}1}$ = *average deviation theory-experiment <2000* cm^{-1} *of all complexes*

Isotope labelling, matrix isolation

The following statements can be made about the spectra shown in Figure 7:

a) CAME, Figure 7a: a full assignment of the experimental peaks as monomer I and II, dimer 1, 2 and 3 and water complex 1, 2 and 3 is discussed in section 2.2.1.

b) CD_3-CAME, Figure 7b: no impurities of CAME are detected and the apparent signals can all be assigned to monomer structure I and II and dimer structures 1, 2 and 3. CAME:water complexes are not detected. The experimental spectrum of CD_3-CAME has the lowest resolution/signal-to-noise-ratio compared to the other spectra which results in a lower number of observed peaks. Especially dimer modes are poorly represented/resolved. As expected the CH-stretching modes above 2550 cm^{-1} "disappear" and shift to wavenumbers below 2300 cm^{-1}. Characteristic hemiester modes of monomer I that are not influenced by substitution of the CH_3/CD_3 group are the ν(OH) mode at 3610 cm^{-1}, ν(C=O) at 1778/1774 cm^{-1}, the δ_{ip}(OH) at1195/1188 and δ_{oop}(CO_3) at 792 cm^{-1}. Unaffected modes of monomer II are ν(C=O) at 1826/1824 cm^{-1} and δ_{ip}(OH) + δ_{ip}(CO_3) at 1337 cm^{-1}. Signals of monomer I that are shifted with a factor between 1.33 and 1.4 are ν_{as}(CD_3) and ν_s(CD_3) modes at 2285, 2280, 2266, 2244, 2101 and 2088 cm^{-1} and δ_{ip}(OH) + δ_{ip}(CO_3) + δ(CH_3) at 988 cm^{-1}. The δ_{ip}(C-O-CD_3) mode is slightly influenced by the isotopic labelling and shifts with a factor of 1.08 to 832 cm^{-1}. Modes that can be assigned for CD_3-CAME but have no equivalent of CAME modes (or could not be detected) for structure I are δ_{ip}(OH) + δ_{ip}(CO_3) at 1401, ν(O-CD_3) at 1115 and 1106 cm^{-1} and δ_s(CD_3) at 905 cm^{-1}. A clearer visualization which modes are affected by the isotopic labelling is displayed and discussed in the context of Figure S7. The observed signals of this experiment do match the positions detected by Reisenauer *et al.*[10].

Dimer signals with a typical H/D shift are not detected, but a clear assignment of all remaining bands is possible. Due to the low signal-to-noise ratio of the CD_3-CAME experiment the OH-stretching modes of dimers are not resolved but the ν(C=O) mode of all three dimers 1, 2 and 3 is observed at 1760 (3), 1758 (2) and 1743 (1) cm^{-1}. Further dimer peaks are the δ_{ip}(OH) + δ_{ip}(CO_3) mode at 1172 (1) and 1460 (2) cm^{-1}, δ_{ip}(OH) at 1321 (2) cm^{-1}, δ_{ip}(OH) + ν(C-OCD_3) at 1321 (1) cm^{-1}, δ_{ip}(CO_3) + ν(C-OCD_3) at 1313 (3) cm^{-1}, ν(O-CD_3) at 1128 (3, 1) cm^{-1}, δ_{oop}(OH) at 1007 (2) $cm^{-1}$and δ_{oop}(CO_3) at 861 (3) cm^{-1}.

c) OD-CAME, Figure 7c: The spectrum of OD came indicates an impurity ratio of 1:1 with non-labeled CAME molecules. All bands that match the pure CAME spectrum are marked with * in the assignment in Table 4 and Table 6. Furthermore, for a suitable assignment of the OD-CAME spectrum also mixed dimers of OD/OH species need to be taken into account and are listed in Table S4 (dimer structures 1, 2 (2_1 and 2_2) and 3). OH/OD exchange leads to a significant shift with a factor of 1.35 of the ν(OD) mode to 2665/2663 cm^{-1} for monomer I

and 2660 cm^{-1} for monomer II. Remarkable peaks appear also for dimer ν(OD) modes at 2229 (1), 2196 (2) and 2126 (3) cm^{-1} with a H/D shift of 1.33 compared to the CAME dimers. Apart from the peaks of pure CAME species, various additional peaks in the regions above 2550 and below 2300 cm^{-1} can be explained as ν(OH/OD), δ_{ip}(OH/OD) and ν(OH/OD) modes of mixed dimers at 2888 (2_1), 2968 (2_2), 2771 (1), 2215 (1), 2207 (1), 2172 (2_2), 2155 (2_1) and 2089 (3) cm^{-1}. The strong ν(C=O) signals of OD-CAME monomer structures I and II are detected at 1774/1770 and 1822/1818 cm^{-1}. The ν(C=O) modes of all OD-dimers and the OD/OH dimers overlap to a broad peak at ~1717 cm^{-1}. In the spectral region between 1490 and 1260 cm^{-1} a high density of bands is observed, which results from monomer and dimer bands of OD-CAME and the CAME impurity as well as numerous bands of mixed dimers. However, the signal at 1449 cm^{-1} can be assigned to the $\delta_s(CH_3)$ of monomer structure I of OD-CAME. The characteristic signal of the $\delta_{ip}(CO_3)$ of monomer I is a shoulder peak at 1329 cm^{-1} of the δ_{ip}(OH) + $\delta_{ip}(CO_3)$ of the CAME impurity at 1327 cm^{-1}. The $\delta_{ip}(CO_3)$ mode of monomer II is found at 1268 cm^{-1}. Other bands in this range originate from OD/OD dimer modes of $\delta_{ip}(CO_3)$ + $\delta(CH_3)$ and OD/OH dimer modes like $\delta(CH_3)$ + $\delta_{ip}(CO_3)$, $\delta_{ip}(CO_3)$, δ_{ip}(OH/OD) + $\delta_{ip}(CO_3)$ and $\delta_{ip}(CO_3)$ (detailed assignment see Table S4). A number of bands near the δ_{ip}(OH) peak of monomer I of the CAME impurity at 1182 cm^{-1} are mainly $\delta(CH_3)$ modes of (mixed) dimers. The δ_{ip}(OH) signal of monomer I is not directly related to the δ_{ip}(OD) mode because the latter is coupled to a ν(C-OD) mode and is found at 1016/1014 cm^{-1} and coupled to $\delta_{ip}(C\text{-}O\text{-}CH_3)$ at 850 cm^{-1} in the OD-CAME spectrum, resulting in shifts of 1.16 and 1.39. The δ_{ip}(OD) + $\delta_{ip}(C\text{-}O\text{-}CH_3)$ of monomer structure II is detected at 865 cm^{-1}. Besides some small dimer peaks (for details see Table 6 and Table S4) remaining assignable peaks are $\nu(O\text{-}CH_3)$ + ν(C-OD) at 1080/1079 cm^{-1} and $\delta_{oop}(CO_3)$ at 794 cm^{-1} of monomer structure I and $\delta_{oop}(CO_3)$ at 786 cm^{-1} of monomer structure II.

d) ^{13}C-CAME, Figure 7d: The amount of CAME impurity in ^{13}C-CAME is much lower than for the OD-CAME experiment. Evaluated from the very small monomer peaks of CAME structure I a ratio of 14 : 1 of ^{13}C : ^{12}C is estimated and used for the intensity adaption of the calculated line spectra displayed in Figure 7d. Signals for the less frequent monomer structure II of the CAME impurity are hardly detected, but bands of mixed dimers need to be taken into account for a complete discussion (dimer structures 1, 2 (2_1 and 2_2) and 3). All bands that match the pure CAME spectrum are marked with * in the assignment in Table 4 and Table 6. An additional experiment after UV-irradiation (see section 2.5.) verifies the assignment of ^{13}C-CAME monomer structures and all bands confirmed by the difference spectrum in Figure 8 are indicated in bold in Table 4. Just like for the difference spectrum of CAME after UV-irradiation also for ^{13}C-CAME UV-irradiation induces a transition of monomer structure I to

II but no bands of dimers (or water complexes) are apparent. Modes that shift by $^{13}C/^{12}C$ substitution with a factor of ~1.03 are ν(C=O) at 1735/1733 and 1790/1787 cm^{-1} of monomer I and II, 1703 1697 and 1685 cm^{-1} of dimer 3, 2 and 1, δ_{ip}(OH) + δ_{ip}(CO_3) + δ(CH_3) at 1362 and 1309 cm^{-1} of monomer I and II, 1478 and 1430 cm^{-1} of dimer 3 and 1 and δ_{oop}(CO_3) at 770 and 762 of monomer I and II and 783 and 772 cm^{-1} of dimer 1/2/3 and 2. Bands of monomer structure I of ^{13}C-CAME that are at the almost same position as for the non-labeled CAME are the ν(OH) mode at 3610 cm^{-1} (3602 cm^{-1} for monomer structure II) and ν(CH_3) modes at 3041, 3005 and 2916 cm^{-1}. This also applies for dimer peaks of ν(OH) + ν(CH_3) and solely ν(OH) at 3017 (1), 3005 (1) and 2930 (2) cm^{-1}. The strong signal of the δ_{ip}(OH) mode of monomer I is detected at 1175/1174 cm^{-1} and by comparison with the spectra after UV-irradiation small signals at 1170 and 1070 cm^{-1} can be assigned to the δ_{ip}(OH) and ν(O-CH_3) modes of monomer II. Further signals are assigned as δ_s(CH_3) of monomer structures at 1447 (I) and 1442 (II) cm^{-1} and δ_{ip}(C-O-CH_3) at 896 (I) and 890 (II) cm^{-1}. Various bands around 1450, 1290 and 1080 cm^{-1} originate from δ_{ip}(CO_3) + δ_{ip}(OH) + δ(CH_3) and ν(O-CH_3) modes of all dimers (for details see Table 6). Besides the small amount of CAME impurity the most apparent signals for mixed dimers are ν(C=O) modes around 1730 cm^{-1} but also small bands above 1300 cm^{-1} (for details see Table S5).

Table S4: Assignment of IR frequencies of OD-OH impurity dimer 1 and 2 (all values in cm^{-1})

**OD-CAME, OH-OD-Dimers**		**Assign.**	
Ar	Calc.	Dimer	
2888	3034	2_1	
2868	3009	2_2	ν(OH/OD)
2771	2919	3	
2215	2273	1	δ_{ip}(OH/OD)
2207			
2172	2225	2_2	
2155	2205	2_1	ν(OH/OD)
2089	2144	3	
	1746	3	
1717 broad	1744	2_2	ν(C=O)
	1741	2_1	
1717 broad	1733	1	$\delta_{oop}(CO_3)$
1471	1479	2_2	$\delta(CH_3)$, $\delta_{ip}(CO_3)$
1372	1389	2_2	
1366	1386	1	$\delta_{ip}(CO_3)$
1358	1365	3	
1351	1360	2_1	
1310	1331	2_1	
1304	1325	1	δ_{ip}(OH/OD), $\delta_{ip}(CO_3)$
1292	1319	3	
1280	1310	2_2	$\delta_{ip}(CO_3)$
1204, 1200	1206, 1207	1, 2_1,	$\delta(CH_3)$
1186	1195	2_2	
		3	
	1084, 1102	1	$\nu(O\text{-}CH_3)$, δ_{oop}(OH/OD)
1075, 1065, 1042, 1023/1021	1125, 1107, 1066	2_1	$\nu(O\text{-}CH_3)$, $\nu(O\text{-}CH_3)$, δ_{ip}(OH/OD)
	1086, 1106, 1118	2_2	$\nu(O\text{-}CH_3)$, $\nu(O\text{-}CH_3)$, δ_{ip}(OH/OD)
	1127, 1118, 1070, 1007	3	$\nu(O\text{-}CH_3)$, $\nu(O\text{-}CH_3)$, δ_{ip}(OH/OD), δ_{oop}(OH/OD)
$\bar{x}_{th\text{-}exp >2000 cm\text{-}1}$	151.5		
$\bar{x}_{th\text{-}exp <2000 cm\text{-}1}$	21.9		

Factor calc. = 0.98

ν = stretching mode, δ = bending mode, ip = in plane, oop = out of plane

$\bar{x}_{th\text{-}exp >2000 cm\text{-}1}$ = *average deviation theory-experiment >2000 cm^{-1} of all dimers*

$\bar{x}_{th\text{-}exp <2000 cm\text{-}1}$ = *average deviation theory-experiment <2000 cm^{-1} of all dimers*

Table S5: Assignment of IR frequencies of ^{13}C-^{12}C impurity dimer 1 and 2 (all values in cm^{-1})

**^{13}C-CAME, ^{13}C-^{12}C-Dimers**		**Assign.**	
Ar	Calc.	Dimer	
2930	3083	2_1, 2_2	ν(OH)
1740	1737	2_1	ν(C=O)
1728, 1725	1721	2_2	
1478	1489	1	δ(CH_3)
	1494	2_2	
1465	1485	2_1	$δ_{ip}$(CO_3), $δ_{ip}$(OH), δ(CH_3)
1430	1437	2_1	$δ_{ip}$(OH), δ(CH_3)
	1436	2_2	
1327	1339	1	$δ_{ip}$(CO_3), $δ_{ip}$(OH)
broad 1299-1281	1332	2_2	
(max. = 1286)	1325	1	
	1319	3	
	1312	2_1	
	1298	3	
	1290	2_1	
783	776	2_1,	$δ_{ip}$(CO_3), $δ_{ip}$(OH)
$\overline{x}_{th\text{-}exp <2000\ cm\text{-}1}$	13.9		

Factor calc. = 0.98
ν = stretching mode, δ = bending mode, ip = in plane
$\overline{x}_{th\text{-}exp <2000\ cm\text{-}1}$ = *average deviation theory-experiment <2000 cm^{-1} of all dimers*

Comparison of CAME, CA and CAEE

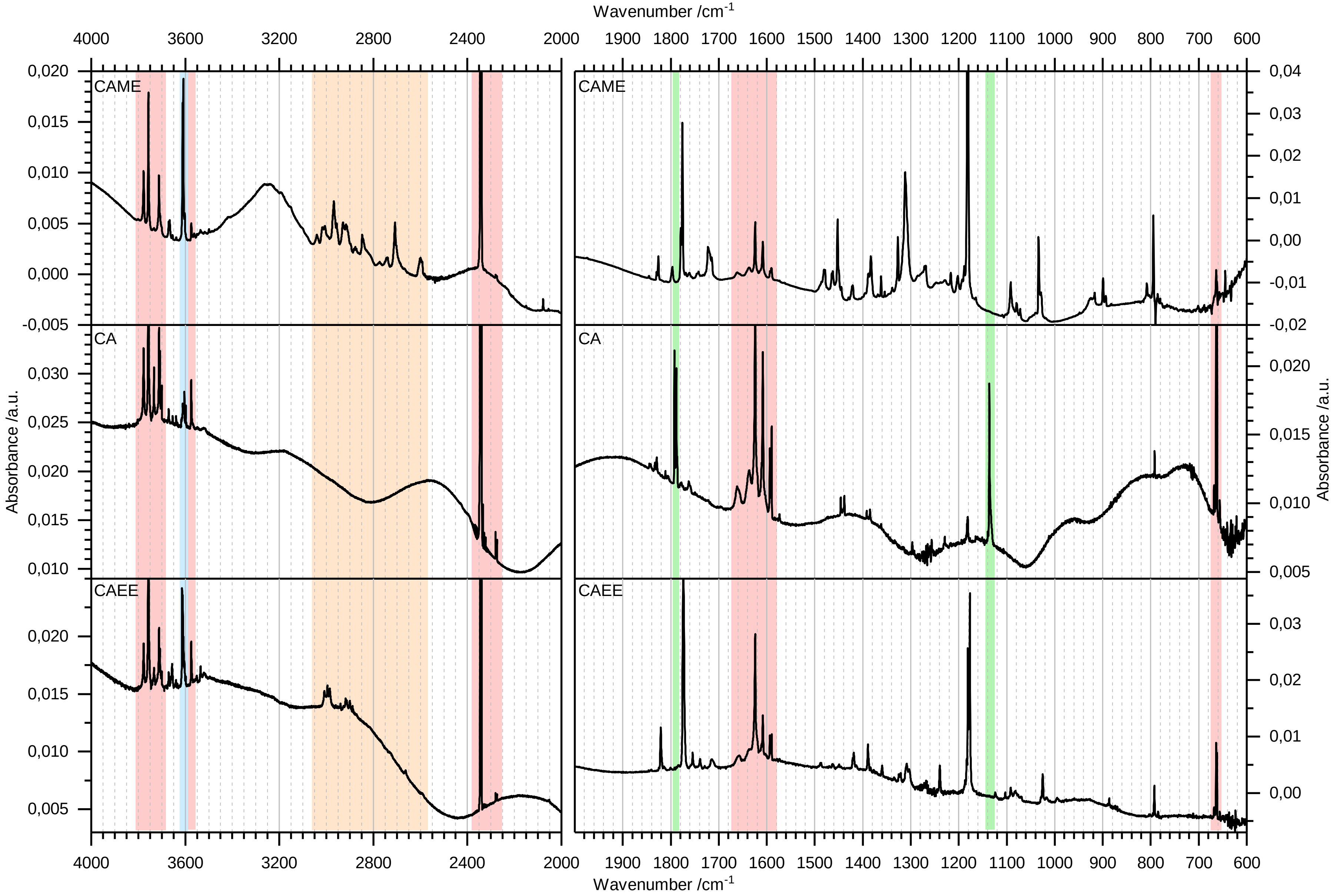

Figure S6: Matrix isolation spectra of a) CAME, b) carbonic acid and c) CAEE. The OH signal is labeled in blue, the area of CH_3-modes and (CAME-)dimer OH-bands is labeled in orange and the two strongest peaks of carbonic acid are marked in green. Spectral regions of H_2O and CO_2 impurities are indicated in red.

Comparison of matrix and solid spectra

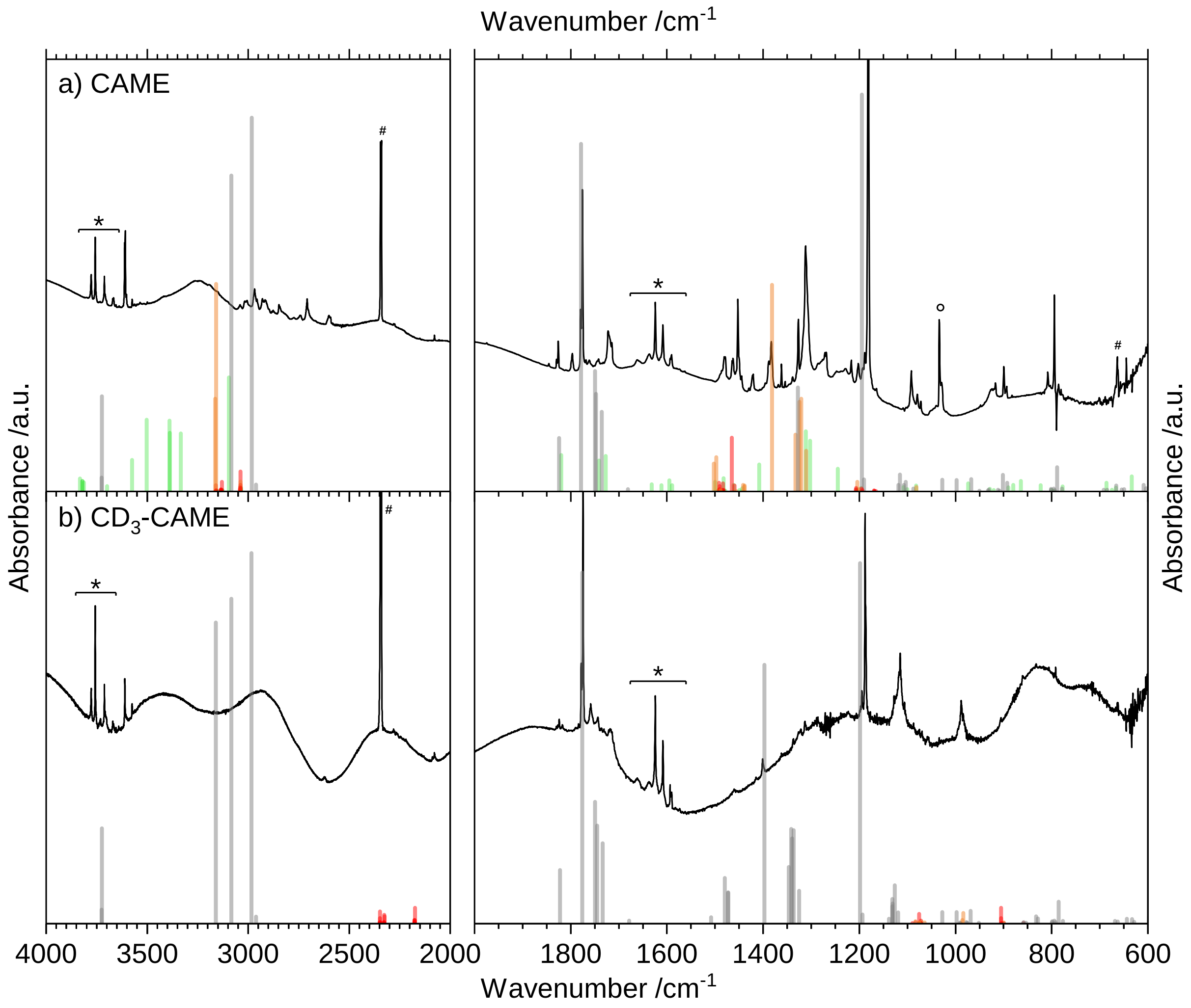

Figure S7: Matrix isolation IR spectra of a) CAME, b) CD_3-CAME. Color code of the MP2/aug-cc-pVTZ calculated spectra for a) and b) is red = pure CH_3/CD_3 vibrations, grey = non-CH_3 vibrational modes/vibrational modes that are not coupled to CH_3/CD_3 vibrations, orange = vibrational modes including CH_3/CD_3 and non-CH_3 vibrations of both monomers I and II and dimers 1, 2 and 3, and green = water complexes 1, 2 and 3. The intensity of the calculated line spectra are the following: int(monomer I)*1, int(monomer II)/6, int(dimer 1, 2 and 3)/10 and int(water complexes 1, 2 and 3)/10. Bands corresponding to CO_2, H_2O and MeOH are labeled with *,$^{\#}$ and ^{o}. Calculated frequencies are scaled by a factor of 0.98.

Evaluation of the composition in matrix and solid

The abundance of structure I in the matrix is lower than in thermodynamic equilibrium. This might indicate that not only structure I sublimates from the solid CAME surface, but also structure II and/or structure III, which then convert during flight to structure I. Assuming that structure III is of no relevance the ratio of structures I : II that sublimes from the surface can be roughly estimated on the basis of the rotational barrier 36.5 kJ mol^{-1} given in Figure 1b. At 210 K sublimation temperature this implies a half-life $\tau(II \rightarrow I)$ against isomerization during the flight time of the molecules in gaseous state of ~ 195 µs. As explained in detail in reference[9], these calculations are based on the Eyring theory and the quasi-equilibrium assumption of transition state theory ($k = \frac{k_B T}{h} \cdot exp\left\{\frac{-\Delta G'}{RT}\right\}$, $\tau = \frac{\ln(2)}{k}$). In 0.5 ms flight time ~2.6 half-lives elapse until the molecule lands in the matrix with a temperature of 10 K. After 2.6 half-lives about 83 % of the molecules with the initial structure II are converted to structure I. The lower barrier for the structure III to structure I isomerization implies that structure III converts much faster, experiencing many half-lives in 0.5 ms. That is, if structure III evaporated from the solid it would convert almost entirely (>99%) to structure I in 0.5 ms in the gas phase, so that at most traces can be found – in agreement with the experimental observation.

Consequently, the ratio observed in the matrix does not directly represent the one in the solid state after sublimation where CAME molecules in the crystal can be forced to be in an unfavorable conformational state because of the crystal field. Once the molecules are frozen in the matrix at a temperature of 10 K no changes should occur during the measurement time of a few minutes. For carbonic acid, it was found that tunneling between two conformers takes a half-life of approximately 4-20 hours[11]. Derived from the considerations above the observed ratio of 6 : 1 for structure I and II in the matrix indicates an original ratio of 1 : 2 sublimating from the crystal. It is impossible to infer whether structure III evaporates and transforms to structure I or does not evaporate at all.

Following an alternative approach, the ratio of the two components before evaporation can be derived from a simple kinetic expression according to the reaction scheme, where x is the fraction of structure II and z of structure I:

$$x \underset{k_2}{\overset{k_1}{\rightleftharpoons}} z \quad ; \quad z = 1 - x$$

In thermodynamic equilibrium, for time $t \to \infty$, $\frac{z}{x} = \frac{k_1}{k_2}$ or $\frac{z}{x} = 22$ (see above), and consequently $k_2 = \frac{k_1}{22}$. For a time of flight of $t_f = 2.6 \times \tau$, where τ is the half-life of structure II, we find $\frac{z}{x} = 6$. Since $k_1 = \frac{\ln 2}{\tau}$, one obtains for the amount x_0 of x at $t = 0$

$$x_0 = \frac{16+7e^{-\beta}}{161e^{-\beta}}$$

with $\beta = \frac{23}{22} k_1 t_f = 1.88$. This finally yields $x_0 = 0.69$ and $z_0 = 0.31$, *i.e.*, a ratio of structure I : II of 1 : 2 before evaporation.

We are aware of the fact that the solid is not built from dimers, but the match of experimental FT-IR spectra of the solid with the calculated spectra of dimers built from structure III is significant in Figure 5 and Figure S3.

References

[1] G. Gattow, W. Behrendt, *Angew. Chem.-Int. Edit.* **1972**, *11*, 534-535.
[2] W. Behrendt, G. Gattow, M. Drager, *Z. Anorg. Allg. Chem.* **1973**, *397*, 237-246.
[3] a) W. Behrendt, G. Gattow, *Z. Anorg. Allg. Chem.* **1973**, *398*, 198-206; b) W. Behrendt, G. Gattow, R. Engler, *Z. Anorg. Allg. Chem.* **1973**, *399*, 193-198.
[4] A. Adam, V. Cirpus, *Z. Anorg. Allg. Chem.* **1994**, *620*, 1702-1706.
[5] K. Winkel, W. Hage, T. Loerting, S. L. Price, E. Mayer, *J. Am. Chem. Soc.* **2007**, *129*, 13863-13871.
[6] W. Hage, A. Hallbrucker, E. Mayer, *J. Chem. Soc. Farad. Trans.* **1996**, *92*, 3183-3195.
[7] K. Nakamoto, Y. A. Sarma, H. Ogoshi, *J. Chem. Phys.* **1965**, *43*, 1177-1181.
[8] a) S. Abouelhassan, F. Salman, M. Elmansy, E. Sheha, *Surf. Rev. Lett.* **2004**, *11*, 83-86; b) J. O. Thomas, R. Tellgren, I. Olovsson, *Acta Crystallogr. Sect. B-Struct. Sci.* **1974**, *30*, 1155-1166.
[9] J. Bernard, E. M. Köck, R. G. Huber, K. R. Liedl, L. Call, R. Schlögl, H. Grothe, T. Loerting, *RSC Adv.* **2017**, *7*, 22222-22233.
[10] H. P. Reisenauer, J. P. Wagner, P. R. Schreiner, *Angew. Chem. Int. Ed.* **2014**, *53*, 11766-11771.
[11] J. P. Wagner, H. P. Reisenauer, V. Hirvonen, C. H. Wu, J. L. Tyberg, W. D. Allen, P. R. Schreiner, *Chem. Commun.* **2016**, *52*, 7858-7861.
